# Does delayed exercise-based priming improve subsequent athletic performance? A systematic review and multilevel meta-analysis

**DOI:** 10.1371/journal.pone.0354720

**Published:** 2026-07-30

**Authors:** Zitao Wang, Haitao Li, Wanyuan Xia, Ziqi Jin, Shiping Fu, Zhenxin Nie

**Affiliations:** 1 School of Physical Education, Yunnan Normal University, Kunming, Yunnan, People’s Republic of China; 2 Wee Kim Wee School of Communication and Information, Nanyang Technological University, Singapore, Singapore; 3 Yunnan Physical Science and Sports Professional College, Kunming, Yunnan, People’s Republic of China; Portugal Football School, Portuguese Football Federation, PORTUGAL

## Abstract

This systematic review and multilevel meta-analysis examined whether delayed exercise-based priming improves subsequent athletic performance. Following PRISMA 2020 principles, 3,095 records were identified; after removing 1,107 duplicates, 1,988 unique records were screened, 212 full-text reports were assessed, 186 reports were excluded at eligibility, and 26 studies were included. Eighteen studies contributed 46 effect sizes to the quantitative synthesis. Risk of bias was assessed with RoB 2 principles calibrated for crossover exercise trials, and certainty was evaluated using GRADE. A three-level random-effects model showed a small positive average effect of delayed priming on subsequent performance (g = 0.232, 95% CI [0.092, 0.372], p = .002), with cluster-robust inference supporting the same conclusion (p = .004). The prediction interval crossed zero (−0.265 to 0.730), and the overall certainty of evidence was low. Delayed exercise-based priming can be used as a practical competition-day strategy when embedded in planned preparation windows, although protocol selection and athlete response should be checked in training before competition use.

## Introduction

In many sports, athletes commonly prepare for afternoon or evening events on the same day as competition. Coaches may use a brief priming session to increase readiness [1], but the same stimulus may also create residual fatigue if the dose or timing is poorly matched. The practical question is therefore not whether exercise can potentiate performance in principle, but whether a low-volume conditioning stimulus performed at least 1 hour earlier can improve later performance without disrupting normal preparation.

Post-activation performance enhancement has been studied extensively in short warm-up-to-performance windows, with mechanistic work emphasizing the balance between potentiation and fatigue [2,3]. Delayed priming asks a different, more applied match-day question: whether an activation session completed hours before performance can still support later readiness. This distinction is important because delayed protocols operate on a longer time scale than traditional PAPE studies and may allow fatigue to dissipate while some neuromuscular readiness is retained [4].

Existing delayed priming evidence spans team sports, aquatic sports, running, and resistance-trained cohorts. Some trials have reported later improvements in explosive or sprint-related outcomes after early-day priming in rugby and soccer contexts [5,6]. Other studies have examined sport-specific or upper-body performance outcomes, including swimming, cricket, and resistance-trained tasks [7,8]. Additional running and field-sport evidence broadens the applied setting but also reinforces that protocols, outcomes, and recovery windows vary across studies [9,10]. This breadth is important for practice because teams rarely prepare for a single isolated test; they must plan warm-up, activation, technical rehearsal, and recovery within the same competition day. Many studies also report multiple outcomes from the same participants, so a synthesis that retains outcome-level information while handling dependence is well suited to this applied evidence base.

We therefore synthesized the available evidence using a multilevel model that retained outcome-level information while accounting for dependence within studies. The aim was to estimate the average performance effect and to clarify the conditions under which delayed priming can be trialed within pre-performance preparation.

This review used multilevel modeling, cluster-robust inference, study-level cross-checks, and sensitivity analyses to support a balanced applied interpretation: delayed priming may be useful when tested and monitored within existing training or competition routines, but it should not be treated as a universal prescription.

## Methods

### Design and reporting

The review followed PRISMA 2020 reporting guidance [11] and was prospectively registered in PROSPERO (registration number CRD420261416856). Quantitative analyses were performed in R 4.6.0 using metafor for the primary multilevel model and meta for an independent study-level cross-check [12,13].

### Search strategy and study selection

We searched PubMed, Scopus, Web of Science Core Collection, and Embase on June 5, 2026, for peer-reviewed English-language articles examining delayed exercise-based priming and subsequent athletic or neuromuscular performance. Conference abstracts, dissertations, theses, and non-peer-reviewed reports were excluded because this review prioritized studies with accessible methods, peer-reviewed reporting, and extractable data suitable for quantitative synthesis. This restriction may have omitted some applied sport-performance evidence, but it improved comparability and auditability of the included record set. Google Scholar was searched on June 6, 2026, as a grey-literature source; records were sorted by relevance, and the first 200 results were screened because relevance declines substantially beyond this point in Google Scholar searches. Reference lists of recent reviews and included studies were hand-searched; one additional record was identified this way [14]. Full database-specific search strings, Google Scholar terms, and yield counts are provided in S1 File.

Title/abstract screening and full-text eligibility assessment were performed independently by two reviewers. Disagreements at either stage were resolved through discussion with a third reviewer. Data extraction and risk-of-bias judgments were completed by one reviewer and independently verified by a second reviewer, with any discrepancies resolved by consensus.

The final PRISMA source data recorded 3,095 records in the raw EndNote export. After 1,107 duplicate records were removed, 1,988 unique records were screened at title/abstract level. Two hundred twelve reports were assessed at full text. Before the final qualitative synthesis, 186 reports were excluded at eligibility, leaving 26 studies in the qualitative synthesis. Eighteen studies with 46 effect sizes were included in the quantitative synthesis. Exclusions were coded into more granular categories covering population or setting mismatch, non-delayed timing, non-exercise or non-priming intervention, absent eligible comparator, non-performance outcome, publication type, insufficient quantitative information, and other PICOS mismatches; the detailed summary is provided in S2 Table. The narrative-only set comprised eight SMD-incompatible studies. The included study set is documented in the supplementary study-characteristics table and synthesis dataset (S3 Table and S1 Data) [5–10,14–33].

### Eligibility criteria

Eligible studies examined an acute exercise-based priming intervention performed before a later athletic or neuromuscular performance test. Delayed priming was operationally defined as a conditioning stimulus completed at least 1 hour before the subsequent performance test; studies assessing outcomes within minutes of the intervention (immediate post-activation potentiation) were excluded. Participants were athletes, trained individuals, or sport-relevant active cohorts. Eligible designs included randomized, counterbalanced, or crossover trials with a no-priming, passive-rest, standardized warm-up, or comparable control condition. Outcomes included jump/power, strength, sprint or agility, repeated-sprint, running economy, swimming, ball velocity, shooting accuracy, and other sport-specific performance measures.

### Data extraction and effect coding

Studies were eligible for qualitative synthesis if they met the review question. Studies were eligible for quantitative synthesis only when data could be converted to a standardized mean difference with defensible variance information. For each outcome, we recorded study identity, design, population, priming type, control type, interval, outcome domain, means, standard deviations, extraction method, effect direction, and bibliographic metadata. Outcomes where lower values reflected better performance were reverse-coded so that positive Hedges’ g indicated better performance after priming.

### Effect-size computation

The primary metric was Hedges’ g [34]. For crossover studies reporting post-condition means and standard deviations, repeated-measures standardized mean differences were computed as g = J x (Mpriming – Mcontrol)/ SDwithin, where SDwithin = sqrt(SDpriming^2 + SDcontrol^2 - 2rSDprimingSDcontrol) and J is the small-sample correction. Outcomes where lower values indicated better performance were multiplied by −1 before synthesis. This approach standardized effects using the within-participant contrast implied by the paired design; when paired correlations or standard deviations of paired differences were unavailable, r was set to 0.50 for the primary analysis [35,36]. Sensitivity analyses repeated the primary model with assumed correlations of 0.30, 0.50, 0.70, and 0.90. All quantitatively compatible effect sizes meeting the main comparator and design criteria were retained in the primary multilevel model; a sensitivity analysis retained one preferred outcome per study, prioritizing sport-specific or primary performance outcomes and then explosive or speed-related outcomes when no single primary outcome was prespecified. Studies with non-convertible percentage change, model-derived marginal effects, medians, or missing variance information were retained narratively, and extractability decisions are summarized in S4 Table.

### Statistical analysis

The primary analysis used a three-level random-effects model estimated with restricted maximum likelihood, with effect sizes nested within studies (rma.mv: yi, vi, random = ~ 1 | study_id/effect_id). This approach is recommended when meta-analyses retain multiple dependent effect sizes from the same study rather than reducing each study to a single outcome [37]. Related multilevel meta-analysis guidance also supports this structure for dependent effects nested within studies [38,39]. Level 2 represented within-study outcome-level heterogeneity and level 3 represented between-study heterogeneity. Heterogeneity and interpretation of dispersion were summarized using Q, variance components, multilevel I^2^, and prediction intervals [40,41]. Cluster-robust variance estimation was used as a small-sample inference check at the study level [42,43]. Prediction intervals and certainty ratings were interpreted alongside the average effect to avoid translating a statistically positive mean effect into an overly general practical recommendation.

### Risk of bias assessment

Risk of bias was assessed using RoB 2 principles, with additional crossover-specific signaling questions for period, carryover, and washout concerns [44,45]. Six domains were judged: D1 = randomization/period-order information; D2 = deviations from intended interventions; D3 = missing outcome data; D4 = outcome measurement; D5 = selection of the reported result; and D6 = crossover-specific carryover/washout/period effects. Quantitative extractability was recorded separately as a reporting-completeness indicator and was not used as a risk-of-bias domain. Overall judgments were classified as low risk, some concerns, or high risk.

### GRADE assessment

The certainty of evidence was evaluated using the GRADE approach [46,47]. Randomized and crossover trials were initially rated as high certainty and then downgraded for risk of bias, inconsistency, indirectness, imprecision, and publication bias. Certainty ratings were assigned for the overall athletic-performance effect, the main outcome-domain subgroup estimates, and the >=6 h interval analysis.

## Results

### PRISMA flow and study selection

**Fig 1:**
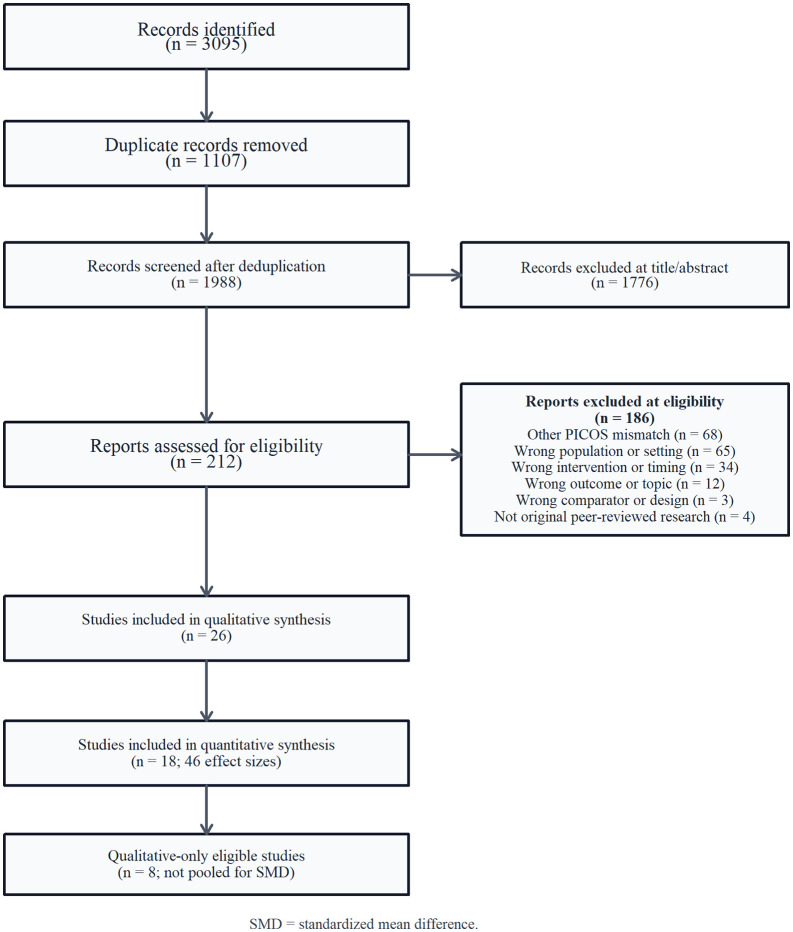
PRISMA 2020 flow diagram. Full-text exclusions were grouped as other PICOS mismatch (n = 68), wrong population or setting (n = 65), wrong intervention or timing (n = 34), wrong outcome or topic (n = 12), wrong comparator or design (n = 3), and not original peer-reviewed research (n = 4). The current formal synthesis includes 26 qualitative studies, of which 18 studies (46 effect sizes) entered the quantitative meta-analysis and 8 were retained narratively only because they were SMD-incompatible. Detailed exclusion categories are provided in S2 Table.

The formal PRISMA dataset began with 3,095 records in the raw EndNote export. After 1,107 duplicate records were removed, 1,988 unique records were screened at title/abstract level. Two hundred twelve reports were assessed at full text; 186 reports were excluded at eligibility, and 26 studies were included in the qualitative synthesis (Fig 1). Eighteen studies with 46 effect sizes were included in the quantitative synthesis. The narrative-only set comprised eight SMD-incompatible studies. The included study set is documented in the supplementary study-characteristics table and synthesis dataset (S3 Table and S1 Data) [5–10,14–33]. The qualitative evidence showed several favorable signals alongside context-specific responses, supporting the value of a quantitative synthesis focused on both average effect and implementation conditions.

### Primary meta-analysis

**Fig 2:**
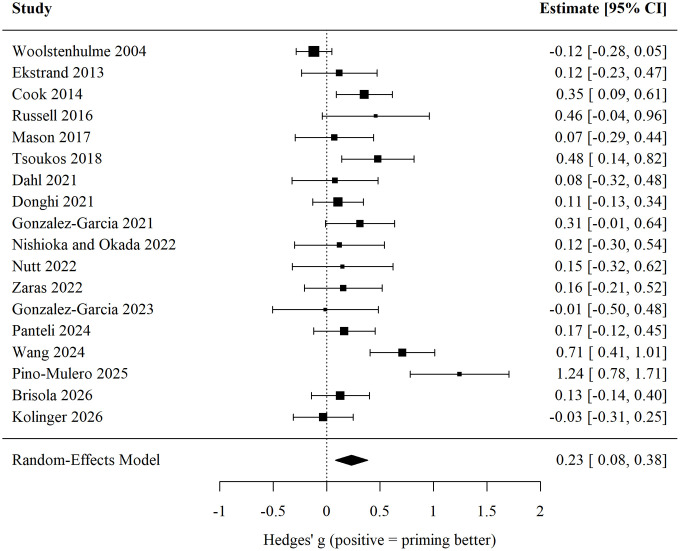
Study-level forest plot for the effect of delayed exercise-based priming on subsequent athletic performance. Positive Hedges’ g values favor priming. The diamond represents the study-level random-effects estimate.

Delayed priming produced a small positive average effect on subsequent performance. The three-level random-effects model yielded Hedges’ g = 0.232, 95% CI [0.092, 0.372], p = .002 (Fig 2). Cluster-robust small-sample inference supported the same conclusion (robust p = .004). The prediction interval ranged from −0.265 to 0.730, indicating meaningful context- and athlete-dependent response variability. For practice, this variability supports a monitored implementation model: coaches can test delayed priming in training, identify responsive athletes and event demands, and then apply the strategy selectively when competition-day logistics allow sufficient recovery.

Heterogeneity was statistically detectable, Q(45) = 65.28, p = .026. The multilevel I^2^ estimate was 46.8%, with heterogeneity concentrated at the between-study level (tau^2^ level 3 = 0.056) rather than within-study outcome-level variation (tau^2^ level 2 < 0.001). A study-level cross-check produced the same pooled point estimate (SMD = 0.233, 95% CI 0.082 to 0.385, p = .0047), with higher study-level I^2^ of 68.7%. The main model, variance components, prediction interval, and cluster-robust result are summarized in Table 1.

**Table 1:** Main model and sensitivity analyses.

Analysis	k	m	g	95% CI	p	PI	tau^2^ L2	tau^2^ L3	I^2^	CR2 p
Main formal model	18	46	0.232	0.092, 0.372	.002	−0.265, 0.730	<0.001	0.056	46.8%	.004
>=6 h only	12	32	0.288	0.086, 0.490	.007	−0.348, 0.924	NA	NA	NA	.014
One preferred outcome per study	18	18	0.258	0.111, 0.404	.002	−0.081, 0.596	NA	NA	NA	.002

Note. k = number of studies; m = number of effect sizes; CR2 = cluster-robust small-sample inference; PI = prediction interval; tau^2^ L2 = within-study variance component; tau^2^ L3 = between-study variance component.

### Sensitivity and subgroup analyses

Exploratory subgroup estimates were most consistently positive for explosive or speed-related outcomes and for sprint-based priming protocols in the available sample (Table 1). These patterns are useful for applied planning because they point toward the types of performance tasks and priming formats most likely to warrant trialing in training. Moderator tests for outcome domain, priming type, and interval were not statistically significant, and the significant control-type moderator test suggests that comparator design may influence observed variation. Leave-one-study-out analyses showed that the pooled study-level estimate remained positive after omitting each study in turn (range g = 0.183 to 0.261; S6 Table).

### Publication bias

**Fig 3:**
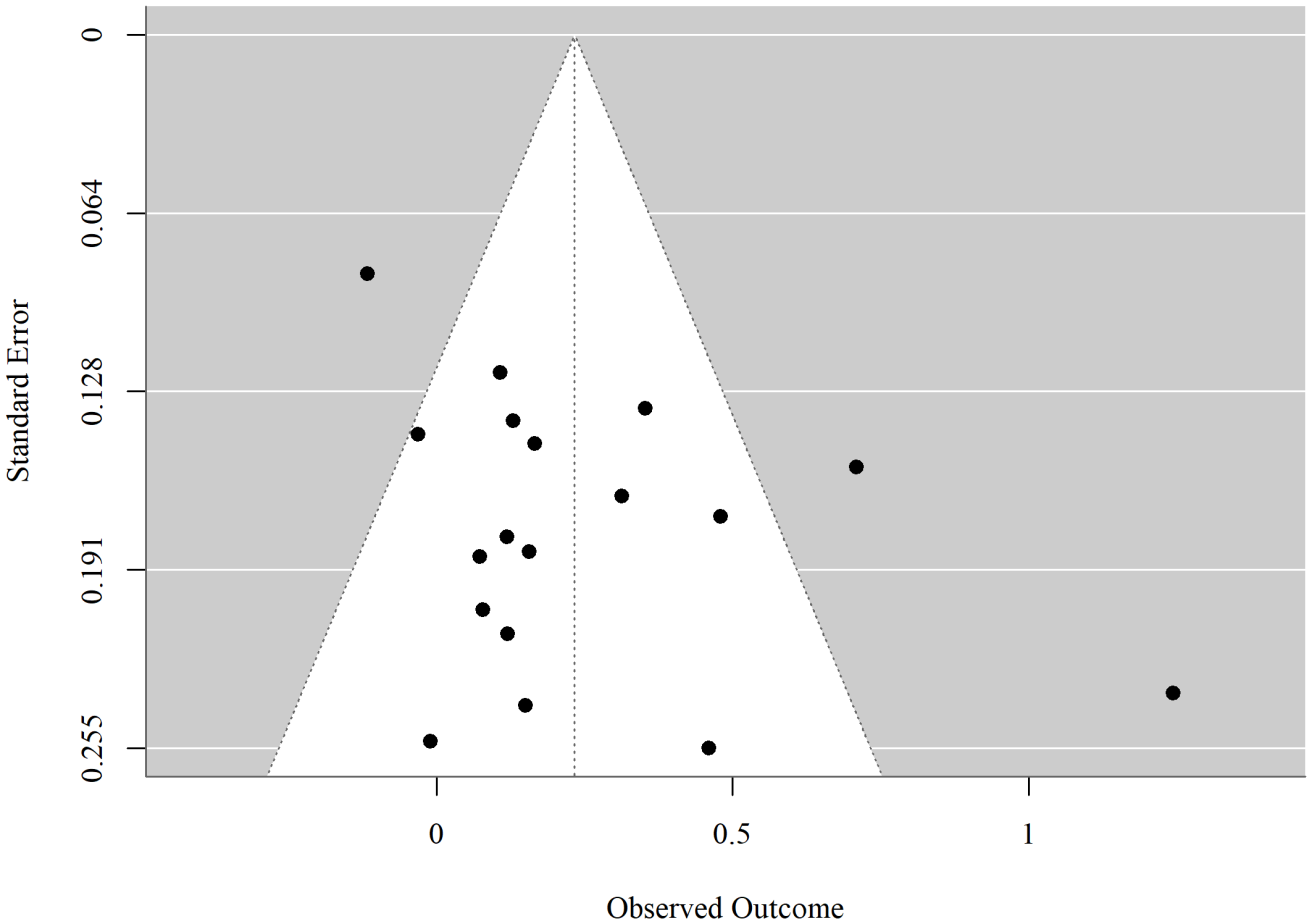
Study-level funnel plot for publication-bias assessment. Each point represents one study-level aggregated effect estimate; the vertical reference line indicates the pooled study-level estimate. Egger-type regression did not indicate statistically significant asymmetry, but the assessment should be interpreted cautiously because only 18 quantitative studies were available.

The study-level funnel plot did not show statistically significant asymmetry (Fig 3). Egger-type regression on aggregated study-level effects gave t = 1.015, df = 16, p = .325. This result is consistent with the overall positive synthesis, although the number of quantitative studies remains modest for small-study effect assessment [48].

### Risk of bias

**Fig 4:**
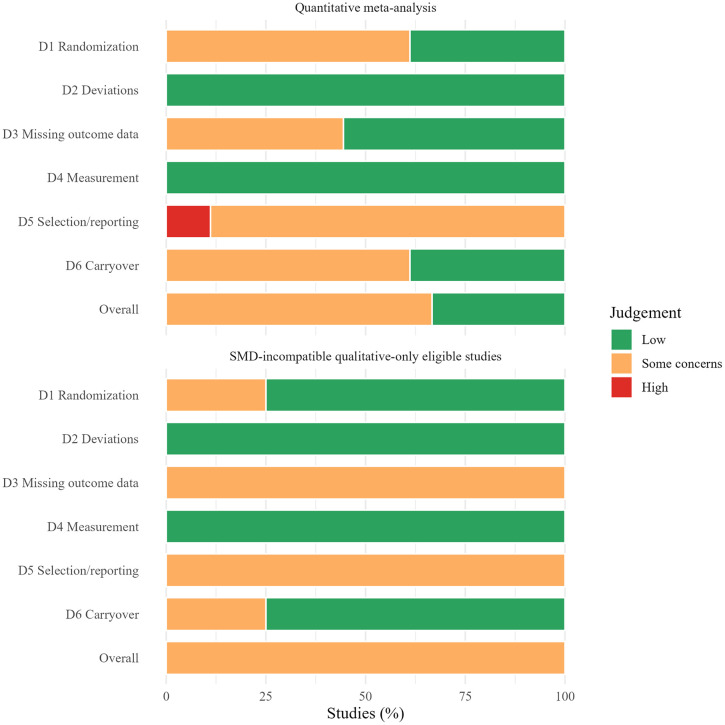
Risk-of-bias summary. Domain-level RoB 2-informed judgments are shown separately for quantitative meta-analysis studies and SMD-incompatible qualitative-only eligible studies. Quantitative extractability was recorded separately and was not treated as a risk-of-bias domain.

Risk-of-bias judgments were completed for all 26 studies in the qualitative synthesis, and the overall summary is shown in Fig 4 and Table 2. Among the 18 studies included in the quantitative meta-analysis, 6 were judged low risk overall and 12 were judged as having some concerns; none was judged high risk overall. The 8 SMD-incompatible qualitative-only studies were judged as having some concerns overall rather than high risk, because non-convertible reporting was treated as a quantitative extractability limitation rather than direct evidence of biased intervention effects. Outcome measurement was generally low risk because performance endpoints were objective.

**Table 2:** Risk-of-bias overall summary.

Synthesis	Low	Some concerns	High
Quantitative meta-analysis studies	6	12	0
SMD-incompatible qualitative-only studies	0	8	0

Note. D1 = randomization/period order; D2 = deviations from intended interventions; D3 = missing outcome data; D4 = outcome measurement; D5 = selection of the reported result; D6 = carryover/washout/period concern. Quantitative extractability and comparator/design compatibility were recorded separately so that SMD incompatibility alone was not treated as high internal bias.

Some-concerns judgments in the quantitative meta-analysis mainly reflected incomplete reporting of sequence generation, period-order/carryover safeguards, prespecified primary outcomes, or variance information. This profile indicates that the evidence base is not dominated by high-risk studies and can support applied interpretation, while also identifying the reporting details that future trials should strengthen.

### Certainty of evidence (GRADE)

Using GRADE, the certainty of evidence for the overall effect on athletic performance was rated low. The main reasons were contextual variability across studies, incomplete reporting of crossover-specific design details, and the exploratory nature of subgroup analyses. A concise GRADE summary is provided in Table 3, and the full supporting table is provided in S5 Table. This rating supports a practical implementation approach based on trialing, monitoring, and athlete-specific decision-making rather than automatic adoption.

**Table 3:** GRADE summary of findings.

Outcome or analysis	Studies	Effect sizes	Effect estimate	Main reason for downgrade	Certainty
Overall athletic performance	18	46	g = 0.232 (95% CI 0.092 to 0.372); PI -0.265 to 0.730	Serious inconsistency/contextual variability; crossover reporting concerns	Low
Direct mean/SD-only evidence	13	37	g = 0.105 (95% CI 0.005 to 0.205)	Imprecision and reduced extractable evidence base	Low
Strength/power outcomes	17	26	g = 0.212 (95% CI 0.097 to 0.326)	Some risk-of-bias concerns and imprecision	Low
Speed/agility outcomes	7	9	g = 0.377 (95% CI -0.025 to 0.778)	Inconsistency and imprecision	Very low
Sport-specific outcomes	8	11	g = 0.075 (95% CI -0.117 to 0.268)	Indirectness and imprecision	Very low
Intervals of at least 6 h	12	32	g = 0.288 (95% CI 0.086 to 0.490); PI -0.348 to 0.924	Serious inconsistency/contextual variability	Low

Note. GRADE = Grading of Recommendations Assessment, Development and Evaluation; PI = prediction interval. Subgroup certainty ratings are exploratory.

## Discussion

### Interpretation of findings

The main finding of this review is that delayed exercise-based priming provides a small but practically relevant average benefit for subsequent athletic performance. The central estimate, Hedges’ g = 0.232, represents an inclusive pooled estimate based on all eligible extractable data while accounting for within-study dependence. From an applied perspective, this finding suggests that delayed priming can be used as an operational strategy to refine competition-day preparation, rather than being viewed only as an acute ergogenic phenomenon tested under laboratory conditions. For example, in a team that already schedules a morning activation, technical rehearsal, or low-load preparation session before an afternoon or evening match, coaches could embed a familiar low-volume priming stimulus into the existing routine while preserving sufficient recovery time before performance. In this sense, delayed priming is not an additional training burden, but a testable, monitorable, and individualized adjustment to the structure of pre-competition preparation.

A plausible explanation is that delayed priming reflects a readiness-fatigue balance supported by several short-term adaptive processes. Low-volume resistance, sprint, or ballistic stimuli may increase motor-unit recruitment efficiency, neural drive, muscle-tendon stiffness, and readiness for explosive force production. At the same time, a recovery interval of several hours may allow acute fatigue to dissipate while retaining part of the activation response. This combination of retained activation and reduced fatigue is particularly relevant for explosive, sprint, jump, and short-duration high-power tasks. By contrast, more complex sport-specific outcomes may be more strongly affected by technical consistency, tactical context, and measurement variability, which may explain why their effects were less clear than those observed for speed- and power-related outcomes.

Although the pooled effect was small, small changes can be meaningful in competitive sport when an intervention is low-cost, familiar, and unlikely to disrupt normal preparation [49,50]. For athletes who already complete morning preparation before afternoon or evening competition, a low-volume priming session may fit the existing schedule without substantial logistical cost. The practical question is therefore whether the expected benefit outweighs the individual athlete’s fatigue risk, not whether the average effect is large in standardized units.

The prediction interval highlights meaningful contextual variability, which is useful for implementation because it points toward individualized use rather than a single fixed protocol. Similar issues arise in recent syntheses of heterogeneous non-pharmacological interventions, where cautious translation is needed when intervention definitions and implementation contexts vary [51]. Exploratory meta-analytic work also supports prediction-oriented interpretation when evidence is heterogeneous and intended for applied decision-making [52]. Related systematic-review work also emphasizes that apparently positive findings should still be interpreted through implementation context and certainty of evidence [53]. Evidence from preventive-intervention reviews further illustrates that population, exposure, and protocol differences can shape the practical meaning of a pooled effect [54]. For coaches, the key issue is to identify which athletes, events, and schedules are most likely to benefit from delayed priming. In the available evidence, effects were directionally more favorable when the recovery interval was at least 6 h and when outcomes were explosive or speed-related. This pattern is consistent with applied training logic: a brief, low-volume, high-quality stimulus may improve neuromuscular readiness before later performance without imposing a substantial fatigue burden.

The risk-of-bias findings indicate that the current evidence base has reporting and design limitations, but remains interpretable and usable for applied decision-making. No study in the quantitative meta-analysis was judged to be at high overall risk of bias, and most studies used crossover or controlled designs with objective performance outcomes. These features provide a reasonable basis for practical trialing while also showing where future confirmation should be strengthened. Some eligible studies could not be synthesized quantitatively because variance information or reporting formats were insufficient, which highlights the need for future studies to report paired differences, within-participant correlations, trial order, and prespecified primary outcomes more completely. Such reporting would improve the usability of delayed priming evidence across sports and competition schedules.

### Practical implications

In practice, delayed priming should first be tested during training or simulated competition days rather than adopted automatically before important competition. Coaches may prioritize familiar, low-volume protocols and explosive or speed-related outcomes, where the current evidence is most promising. Athlete response, sport demands, recovery interval, and fatigue state should guide whether the strategy is retained for competition use.

### Strengths and limitations

Several limitations should be acknowledged without obscuring the practical signal observed in the evidence. Many included studies used crossover designs but incompletely reported paired-condition correlations or paired-difference standard deviations, which affected the precision of repeated-measures effect-size estimation. The inclusion of multiple outcomes per study required multilevel modeling, and subgroup analyses remained limited by the number of available studies. Some relevant studies could not be meta-analyzed because variance data were insufficient or outcomes were reported in non-extractable formats. These limitations mainly indicate where future sport-performance trials should improve reporting and design standardization, while the present synthesis still provides a usable evidence base for carefully monitored applied trialing.

## Conclusion

Delayed exercise-based priming was associated with a small positive average effect on subsequent athletic performance. Current evidence supports individualized testing during training, especially for familiar low-volume protocols separated from performance by sufficient recovery time and for explosive or speed-related outcomes. Because the certainty of evidence is low and effects vary across contexts, delayed priming should be implemented with athlete-specific monitoring rather than as a universal competition-day prescription.

## Supporting information

S1 Checklist PRISMA 2020 checklist. (DOCX)

S1 File Database-specific search strategy and source counts. (XLSX)

S1 Data Source data files used to reproduce the quantitative analyses, tables, and figures. (ZIP)

S1 Code R analysis code used to reproduce the quantitative analyses, tables, and figures. (ZIP)

S2 Table Full-text exclusion categories. (DOCX)

S3 Table Characteristics of studies included in the quantitative meta-analysis. (DOCX)

S4 Table Effect-size reconstruction and extractability decisions. (DOCX)

S5 Table GRADE summary of findings. (DOCX)

S6 Table Leave-one-study-out sensitivity analysis. (DOCX)
