# Supplementary material for: Does delayed exercise-based priming improve subsequent athletic performance? A systematic review and multilevel meta-analysis
Source: PLoS One. 2026 Jul 30;21(7):e0354720. doi: 10.1371/journal.pone.0354720 (PMC13422850; doi:10.1371/journal.pone.0354720)
Supplement: S1 Checklist — (DOCX) [file pone.0354720.s001.docx]

**PRISMA 2020 Checklist**

Manuscript: Delayed Exercise-Based Priming and Same-Day Athletic Performance: A Systematic Review and Multilevel Meta-Analysis

Checklist based on the PRISMA 2020 statement for systematic reviews and meta-analyses.

| **Section/topic** | **Item** | **Checklist item** | **Description** | **Location where item is reported** |
| --- | --- | --- | --- | --- |
| TITLE | 1 | Title | Identify the report as a systematic review. | Title page; manuscript title |
| ABSTRACT | 2 | Abstract | See the PRISMA 2020 for Abstracts checklist. | Abstract |
| INTRODUCTION | 3 | Rationale | Describe the rationale for the review in the context of existing knowledge. | Introduction |
| INTRODUCTION | 4 | Objectives | Provide an explicit statement of the objective(s) or question(s) the review addresses. | End of Introduction |
| METHODS | 5 | Eligibility criteria | Specify the inclusion and exclusion criteria for the review and how studies were grouped for synthesis. | Methods: Eligibility Criteria |
| METHODS | 6 | Information sources | Specify all databases, registers, websites, organisations, reference lists, and other sources searched or consulted; specify the date when each source was last searched or consulted. | Methods: Search Strategy and Study Selection; Supplementary Table S1 |
| METHODS | 7 | Search strategy | Present the full search strategies for all databases, registers, and websites, including any filters and limits used. | Supplementary Table S1; Supplementary Search Strategy spreadsheet |
| METHODS | 8 | Selection process | Specify the methods used to decide whether a study met the inclusion criteria, including how many reviewers screened each record and report, whether they worked independently, and how disagreements were resolved. | Methods: Search Strategy and Study Selection |
| METHODS | 9 | Data collection process | Specify the methods used to collect data from reports, including how many reviewers collected data, whether they worked independently, processes for obtaining or confirming data, and automation tools if used. | Methods: Data Extraction and Effect Coding |
| METHODS | 10a | Data items | List and define all outcomes for which data were sought. Specify whether all results compatible with each outcome domain in each study were sought. | Methods: Data Extraction and Effect Coding; Supplementary Tables |
| METHODS | 10b | Data items | List and define all other variables for which data were sought, such as participant and intervention characteristics, funding sources, and study design. | Methods: Eligibility Criteria; Data Extraction and Effect Coding; Supplementary Tables |
| METHODS | 11 | Study risk of bias assessment | Specify the methods used to assess risk of bias in the included studies, including details of the tools used, how many reviewers assessed each study, whether they worked independently, and how disagreements were resolved. | Methods: Risk of Bias Assessment; Supplementary Table S9 |
| METHODS | 12 | Effect measures | Specify for each outcome the effect measure(s), such as risk ratio or mean difference, used in the synthesis or presentation of results. | Methods: Effect-Size Computation |
| METHODS | 13a | Synthesis methods | Describe the processes used to decide which studies were eligible for each synthesis. | Methods: Eligibility Criteria; Data Extraction and Effect Coding |
| METHODS | 13b | Synthesis methods | Describe any methods required to prepare the data for presentation or synthesis, such as handling missing summary statistics or data conversions. | Methods: Effect-Size Computation |
| METHODS | 13c | Synthesis methods | Describe any methods used to tabulate or visually display results of individual studies and syntheses. | Methods: Statistical Analysis; Results figures and tables |
| METHODS | 13d | Synthesis methods | Describe any methods used to synthesize results and provide a rationale for the choices. If meta-analysis was performed, describe the model, methods to identify heterogeneity, and software used. | Methods: Statistical Analysis |
| METHODS | 13e | Synthesis methods | Describe any methods used to explore possible causes of heterogeneity among study results. | Methods: Statistical Analysis; Sensitivity and Subgroup Analyses |
| METHODS | 13f | Synthesis methods | Describe any sensitivity analyses conducted to assess robustness of the synthesized results. | Methods: Statistical Analysis; Results: Sensitivity and Subgroup Analyses |
| METHODS | 14 | Reporting bias assessment | Describe any methods used to assess risk of bias due to missing results in a synthesis. | Methods: Statistical Analysis; Results: Publication Bias |
| METHODS | 15 | Certainty assessment | Describe any methods used to assess certainty or confidence in the body of evidence for an outcome. | Methods: GRADE Assessment |
| RESULTS | 16a | Study selection | Describe the results of the search and selection process, from the number of records identified to the number of studies included, ideally using a flow diagram. | Results: Study Selection; Figure 1 |
| RESULTS | 16b | Study selection | Cite studies that appeared to meet the inclusion criteria but were excluded, and explain why they were excluded. | Results: Study Selection; Supplementary Tables S2 and S12 |
| RESULTS | 17 | Study characteristics | Cite each included study and present its characteristics. | Results: Study Selection; Table 2; Supplementary Table S3 |
| RESULTS | 18 | Risk of bias in studies | Present assessments of risk of bias for each included study. | Results: Risk of Bias; Figure 3; Supplementary Table S9 |
| RESULTS | 19 | Results of individual studies | For all outcomes, present summary statistics for each study and, where applicable, effect estimates and confidence intervals, ideally using structured tables or plots. | Figure 2; Supplementary Tables S4 and S5 |
| RESULTS | 20a | Results of syntheses | For each synthesis, briefly summarise the characteristics and risk of bias among contributing studies. | Results: Primary Meta-Analysis; Risk of Bias |
| RESULTS | 20b | Results of syntheses | Present results of all statistical syntheses conducted. If meta-analysis was done, present the summary estimate and precision, and measures of heterogeneity. | Results: Primary Meta-Analysis; Table 1; Figure 2 |
| RESULTS | 20c | Results of syntheses | Present results of all investigations of possible causes of heterogeneity among study results. | Results: Sensitivity and Subgroup Analyses; Supplementary Tables |
| RESULTS | 20d | Results of syntheses | Present results of all sensitivity analyses conducted to assess robustness of the synthesized results. | Results: Sensitivity and Subgroup Analyses; Supplementary Tables |
| RESULTS | 21 | Reporting biases | Present assessments of risk of bias due to missing results for each synthesis assessed. | Results: Publication Bias; Supplementary Figure 3 |
| RESULTS | 22 | Certainty of evidence | Present assessments of certainty or confidence in the body of evidence for each outcome assessed. | Results: Certainty of Evidence; Supplementary Table S8 |
| DISCUSSION | 23a | Discussion | Provide a general interpretation of the results in the context of other evidence. | Discussion |
| DISCUSSION | 23b | Discussion | Discuss any limitations of the evidence included in the review. | Discussion: limitations paragraph |
| DISCUSSION | 23c | Discussion | Discuss any limitations of the review processes used. | Discussion: limitations paragraph |
| DISCUSSION | 23d | Discussion | Discuss implications of the results for practice, policy, and future research. | Discussion; Practical Implications; Conclusion |
| OTHER INFORMATION | 24a | Registration and protocol | Provide registration information for the review, including register name and registration number, or state that the review was not registered. | Methods: Design and Reporting |
| OTHER INFORMATION | 24b | Registration and protocol | Indicate where the review protocol can be accessed, or state that a protocol was not prepared. | Methods: Design and Reporting |
| OTHER INFORMATION | 24c | Registration and protocol | Describe and explain any amendments to information provided at registration or in the protocol. | Methods: Design and Reporting |
| OTHER INFORMATION | 25 | Support | Describe sources of financial or non-financial support for the review and the role of the funders or sponsors. | Declarations: Funding |
| OTHER INFORMATION | 26 | Competing interests | Declare any competing interests of review authors. | Declarations: Conflicts of Interest |
| OTHER INFORMATION | 27 | Availability of data, code, and other materials | Report which materials are publicly available and where they can be found. | Declarations: Data and Code Availability; Supplementary Code |
