# Supplementary material for: Does delayed exercise-based priming improve subsequent athletic performance? A systematic review and multilevel meta-analysis
Source: PLoS One. 2026 Jul 30;21(7):e0354720. doi: 10.1371/journal.pone.0354720 (PMC13422850; doi:10.1371/journal.pone.0354720)
Supplement: S1 Data — (ZIP) [file pone.0354720.s003.zip › analysis_code/S4_Analysis_Code.docx]

Supplementary Code

Analysis and figure/table generation scripts for the delayed priming meta-analysis.

Note: This supplementary file contains code only. Data files are not included in this submission package and will be provided through the final repository link when appropriate.

**Included unique scripts:** 10

Analysis code

formal_meta_analysis.R

0001 # Formal meta-analysis for delayed exercise-based priming

0002 # Uses metafor for multilevel models and meta for study-level cross-checks.

0003

0004 options(repos = c(CRAN = "https://cran.rstudio.com/"))

0005 packages <- c(

0006 "metafor", "meta", "clubSandwich", "readr", "dplyr", "tidyr",

0007 "stringr", "ggplot2", "forcats", "purrr", "tibble"

0008 )

0009 missing <- packages[!(packages %in% rownames(installed.packages()))]

0010 if (length(missing) > 0) install.packages(missing, dependencies = TRUE)

0011

0012 library(metafor)

0013 library(meta)

0014 library(clubSandwich)

0015 library(readr)

0016 library(dplyr)

0017 library(tidyr)

0018 library(stringr)

0019 library(ggplot2)

0020 library(forcats)

0021 library(purrr)

0022 library(tibble)

0023

0024 script_arg <- commandArgs(trailingOnly = FALSE)

0025 script_path <- normalizePath(sub("^--file=", "", script_arg[grepl("^--file=", script_arg)][1]), winslash = "/", mustWork = TRUE)

0026 root <- normalizePath(file.path(dirname(script_path), ".."), winslash = "/", mustWork = TRUE)

0027 data_path <- file.path(root, "data", "extracted_data_master.csv")

0028 out_dir <- file.path(root, "analysis", "output")

0029 fig_dir <- file.path(out_dir, "figures")

0030 tab_dir <- file.path(out_dir, "tables")

0031 meta_dir <- file.path(out_dir, "metadata")

0032 log_dir <- file.path(out_dir, "logs")

0033 obj_dir <- file.path(out_dir, "r_objects")

0034 dir.create(fig_dir, showWarnings = FALSE, recursive = TRUE)

0035 dir.create(tab_dir, showWarnings = FALSE, recursive = TRUE)

0036 dir.create(meta_dir, showWarnings = FALSE, recursive = TRUE)

0037 dir.create(log_dir, showWarnings = FALSE, recursive = TRUE)

0038 dir.create(obj_dir, showWarnings = FALSE, recursive = TRUE)

0039

0040 analysis_date <- format(Sys.time(), "%Y-%m-%d %H:%M:%S")

0041

0042 raw <- read_csv(data_path, show_col_types = FALSE) %>%

0043 filter(include_main == 1, direction %in% c("higher_better", "lower_better")) %>%

0044 mutate(

0045 study_id = as.character(study_id),

0046 effect_id = as.character(effect_id),

0047 design = as.character(design),

0048 outcome_domain = as.character(outcome_domain),

0049 priming_type = as.character(priming_type),

0050 control_type = as.character(control_type),

0051 outcome_domain_formal = case_when(

0052 outcome_domain %in% c("jump_power", "strength_power", "strength_force") ~ "strength_power",

0053 outcome_domain %in% c("sprint", "RSA", "COD", "speed_agility") ~ "speed_agility",

0054 outcome_domain %in% c("sport_specific") ~ "sport_specific",

0055 TRUE ~ outcome_domain

0056 ),

0057 priming_group = case_when(

0058 priming_type %in% c("sprint", "resisted_sprint") ~ "sprint_based",

0059 priming_type %in% c("ballistic") ~ "ballistic_jump",

0060 TRUE ~ priming_type

0061 ),

0062 interval_group = case_when(

0063 interval_h < 4 ~ "1_to_3h",

0064 interval_h < 6 ~ "4_to_5h",

0065 TRUE ~ "6h_or_more"

0066 )

0067 )

0068

0069 calc_cross_es <- function(df, r = 0.5) {

0070 df %>%

0071 mutate(

0072 sd_within = sqrt(sd_priming^2 + sd_control^2 - 2 * r * sd_priming * sd_control),

0073 d_rm = (mean_priming - mean_control) / sd_within,

0074 J = 1 - (3 / (4 * n_priming - 5)),

0075 yi_calc = J * d_rm,

0076 vi_calc = (1 / n_priming + yi_calc^2 / (2 * n_priming)) * J^2,

0077 es_method = "crossover rm-SMD; assumed r=0.5"

0078 )

0079 }

0080

0081 calc_prepost_es <- function(df, r = 0.5) {

0082 df %>%

0083 mutate(

0084 d_change = (mean_priming - mean_control) / sd_control,

0085 J = 1 - (3 / (4 * n_priming - 5)),

0086 yi_calc = J * d_change,

0087 vi_calc = (2 * (1 - r) / n_priming + yi_calc^2 / (2 * n_priming)) * J^2,

0088 es_method = "pre-post SMD; assumed r=0.5"

0089 )

0090 }

0091

0092 calc_ind_es <- function(df) {

0093 esc <- escalc(

0094 measure = "SMD",

0095 m1i = mean_priming, sd1i = sd_priming, n1i = n_priming,

0096 m2i = mean_control, sd2i = sd_control, n2i = n_control,

0097 data = df

0098 )

0099 df$yi_calc <- esc$yi

0100 df$vi_calc <- esc$vi

0101 df$es_method <- "independent-groups SMD"

0102 df

0103 }

0104

0105 dat_es <- raw %>% mutate(yi = NA_real_, vi = NA_real_, es_method = NA_character_)

0106

0107 reported_idx <- which(!is.na(dat_es$yi_reported) & !is.na(dat_es$vi_reported))

0108 if (length(reported_idx) > 0) {

0109 dat_es$yi[reported_idx] <- dat_es$yi_reported[reported_idx]

0110 dat_es$vi[reported_idx] <- dat_es$vi_reported[reported_idx]

0111 dat_es$es_method[reported_idx] <- "reported ES; approximate variance"

0112 }

0113

0114 remaining <- which(is.na(dat_es$yi))

0115 if (length(remaining) > 0) {

0116 pp_idx <- remaining[grepl("pre-post|repeated measures", dat_es$design[remaining], ignore.case = TRUE)]

0117 if (length(pp_idx) > 0) {

0118 tmp <- calc_prepost_es(dat_es[pp_idx, ], r = 0.5)

0119 dat_es[pp_idx, c("yi", "vi", "es_method")] <- tmp[, c("yi_calc", "vi_calc", "es_method")]

0120 }

0121 remaining <- which(is.na(dat_es$yi))

0122 cross_idx <- remaining[dat_es$design[remaining] == "crossover"]

0123 if (length(cross_idx) > 0) {

0124 tmp <- calc_cross_es(dat_es[cross_idx, ], r = 0.5)

0125 dat_es[cross_idx, c("yi", "vi", "es_method")] <- tmp[, c("yi_calc", "vi_calc", "es_method")]

0126 }

0127 remaining <- which(is.na(dat_es$yi))

0128 if (length(remaining) > 0) {

0129 tmp <- calc_ind_es(dat_es[remaining, ])

0130 dat_es[remaining, c("yi", "vi", "es_method")] <- tmp[, c("yi_calc", "vi_calc", "es_method")]

0131 }

0132 }

0133

0134 dat_es <- dat_es %>%

0135 mutate(

0136 yi = if_else(direction == "lower_better" & is.na(yi_reported), -yi, yi),

0137 vi = as.numeric(vi),

0138 se = sqrt(vi),

0139 ci_lb = yi - 1.96 * se,

0140 ci_ub = yi + 1.96 * se

0141 ) %>%

0142 filter(!is.na(yi), !is.na(vi), vi > 0)

0143

0144 write_csv(dat_es, file.path(tab_dir, "formal_effect_sizes.csv"))

0145

0146 fit_3l <- function(df, mods = NULL) {

0147 if (is.null(mods)) {

0148 rma.mv(yi = yi, V = vi, random = ~ 1 | study_id / effect_id,

0149 method = "REML", test = "t", data = df)

0150 } else {

0151 rma.mv(yi = yi, V = vi, mods = mods, random = ~ 1 | study_id / effect_id,

0152 method = "REML", test = "t", data = df)

0153 }

0154 }

0155

0156 calc_i2 <- function(res, df) {

0157 W <- diag(1 / df$vi)

0158 X <- model.matrix(res)

0159 P <- W - W %*% X %*% solve(t(X) %*% W %*% X) %*% t(X) %*% W

0160 typical_vi <- (nrow(df) - ncol(X)) / sum(diag(P))

0161 total_tau2 <- sum(res$sigma2)

0162 tibble(

0163 typical_vi = typical_vi,

0164 I2_total = 100 * total_tau2 / (total_tau2 + typical_vi),

0165 I2_level3 = 100 * res$sigma2[1] / (total_tau2 + typical_vi),

0166 I2_level2 = 100 * res$sigma2[2] / (total_tau2 + typical_vi)

0167 )

0168 }

0169

0170 summarise_model <- function(label, df) {

0171 if (n_distinct(df$study_id) < 2 || nrow(df) < 2) return(NULL)

0172 res <- fit_3l(df)

0173 pred <- predict(res)

0174 robust <- tryCatch(

0175 coef_test(res, vcov = "CR2", cluster = df$study_id, test = "Satterthwaite"),

0176 error = function(e) tibble(SE = NA_real_, p_Satt = NA_real_)

0177 )

0178 i2s <- calc_i2(res, df)

0179 tibble(

0180 analysis = label,

0181 k_studies = n_distinct(df$study_id),

0182 m_effects = nrow(df),

0183 g = as.numeric(coef(res)),

0184 ci_lb = res$ci.lb,

0185 ci_ub = res$ci.ub,

0186 p = res$pval,

0187 pi_lb = pred$cr.lb,

0188 pi_ub = pred$cr.ub,

0189 tau2_level3 = res$sigma2[1],

0190 tau2_level2 = res$sigma2[2],

0191 I2_total = i2s$I2_total,

0192 I2_level3 = i2s$I2_level3,

0193 I2_level2 = i2s$I2_level2,

0194 Q = res$QE,

0195 Q_p = res$QEp,

0196 robust_se = robust$SE[1],

0197 robust_p = robust$p_Satt[1]

0198 )

0199 }

0200

0201 res_main <- fit_3l(dat_es)

0202 robust_main <- coef_test(res_main, vcov = "CR2", cluster = dat_es$study_id, test = "Satterthwaite")

0203 pred_main <- predict(res_main)

0204

0205 preferred_domain_order <- c(

0206 "sport_specific" = 1,

0207 "speed_agility" = 2,

0208 "strength_power" = 3

0209 )

0210 dat_one <- dat_es %>%

0211 mutate(priority = if_else(outcome_domain_formal %in% names(preferred_domain_order),

0212 preferred_domain_order[outcome_domain_formal], 99)) %>%

0213 arrange(study_id, priority, effect_id) %>%

0214 group_by(study_id) %>%

0215 slice(1) %>%

0216 ungroup()

0217

0218 models <- bind_rows(

0219 summarise_model("Main formal model", dat_es),

0220 summarise_model("Crossover only", filter(dat_es, design == "crossover")),

0221 summarise_model(">=4 h only", filter(dat_es, interval_h >= 4)),

0222 summarise_model("≥6 h only", filter(dat_es, interval_h >= 6)),

0223 summarise_model("One preferred outcome per study", dat_one)

0224 )

0225 write_csv(models, file.path(tab_dir, "overall_and_sensitivity_models.csv"))

0226

0227 subgroup_model <- function(var) {

0228 dat_es %>%

0229 filter(!is.na(.data[[var]])) %>%

0230 group_split(.data[[var]]) %>%

0231 map_dfr(function(df) {

0232 label <- as.character(df[[var]][1])

0233 out <- summarise_model(label, df)

0234 if (is.null(out)) {

0235 w <- 1 / df$vi

0236 tibble(

0237 analysis = label, k_studies = n_distinct(df$study_id), m_effects = nrow(df),

0238 g = sum(w * df$yi) / sum(w),

0239 ci_lb = g - 1.96 * sqrt(1 / sum(w)),

0240 ci_ub = g + 1.96 * sqrt(1 / sum(w)),

0241 p = NA_real_, pi_lb = NA_real_, pi_ub = NA_real_,

0242 tau2_level3 = NA_real_, tau2_level2 = NA_real_,

0243 I2_total = NA_real_, I2_level3 = NA_real_, I2_level2 = NA_real_,

0244 Q = NA_real_, Q_p = NA_real_, robust_se = NA_real_, robust_p = NA_real_

0245 )

0246 } else out

0247 }) %>%

0248 rename(subgroup = analysis) %>%

0249 mutate(subgroup_variable = var, .before = 1)

0250 }

0251

0252 subgroups <- bind_rows(

0253 subgroup_model("outcome_domain_formal"),

0254 subgroup_model("priming_group"),

0255 subgroup_model("interval_group"),

0256 subgroup_model("control_type"),

0257 subgroup_model("athlete_level")

0258 )

0259 write_csv(subgroups, file.path(tab_dir, "subgroup_models.csv"))

0260

0261 moderator_tests <- list(

0262 outcome_domain_formal = ~ factor(outcome_domain_formal),

0263 priming_group = ~ factor(priming_group),

0264 interval_group = ~ factor(interval_group),

0265 control_type = ~ factor(control_type),

0266 athlete_level = ~ factor(athlete_level)

0267 ) %>%

0268 imap_dfr(function(mod, name) {

0269 fit <- tryCatch(fit_3l(dat_es, mods = mod), error = function(e) NULL)

0270 if (is.null(fit)) return(tibble(moderator = name, QM = NA_real_, df = NA_real_, p = NA_real_))

0271 tibble(moderator = name, QM = fit$QM, df = fit$m - 1, p = fit$QMp)

0272 })

0273 write_csv(moderator_tests, file.path(tab_dir, "moderator_tests.csv"))

0274

0275 study_level <- dat_es %>%

0276 group_by(study_id, author, year) %>%

0277 summarise(

0278 yi = weighted.mean(yi, w = 1 / vi),

0279 vi = 1 / sum(1 / vi),

0280 m_effects = n(),

0281 .groups = "drop"

0282 ) %>%

0283 arrange(year, author)

0284 write_csv(study_level, file.path(tab_dir, "study_level_aggregated_effects.csv"))

0285

0286 res_study <- rma(yi, vi, data = study_level, method = "REML", test = "knha")

0287 meta_study <- metagen(

0288 TE = yi, seTE = sqrt(vi), studlab = study_id,

0289 data = study_level, sm = "SMD",

0290 common = FALSE, random = TRUE, method.tau = "REML",

0291 method.random.ci = "HK"

0292 )

0293

0294 sink(file.path(log_dir, "formal_model_output.txt"))

0295 cat("Formal delayed exercise-based priming meta-analysis\n")

0296 cat("Analysis date:", analysis_date, "\n\n")

0297 cat("Studies:", n_distinct(dat_es$study_id), "\n")

0298 cat("Effect sizes:", nrow(dat_es), "\n\n")

0299 cat("metafor three-level model\n")

0300 print(summary(res_main))

0301 cat("\nPrediction interval\n")

0302 print(pred_main)

0303 cat("\nCR2 robust test\n")

0304 print(robust_main)

0305 cat("\nmeta package study-level cross-check\n")

0306 print(summary(meta_study))

0307 cat("\nSensitivity models\n")

0308 print(models)

0309 cat("\nModerator tests\n")

0310 print(moderator_tests)

0311 sink()

0312

0313 saveRDS(list(

0314 dat_es = dat_es, res_main = res_main, robust_main = robust_main,

0315 pred_main = pred_main, study_level = study_level, res_study = res_study,

0316 meta_study = meta_study, models = models, subgroups = subgroups,

0317 moderator_tests = moderator_tests

0318 ), file.path(obj_dir, "formal_meta_objects.rds"))

0319

0320 write_csv(tibble(

0321 item = c("analysis_date", "k_studies", "m_effects", "main_g", "main_ci_lb", "main_ci_ub",

0322 "main_p", "robust_p", "prediction_lb", "prediction_ub"),

0323 value = c(analysis_date, n_distinct(dat_es$study_id), nrow(dat_es),

0324 as.numeric(coef(res_main)), res_main$ci.lb, res_main$ci.ub, res_main$pval,

0325 robust_main$p_Satt[1], pred_main$cr.lb, pred_main$cr.ub)

0326 ), file.path(tab_dir, "formal_key_results.csv"))

0327

0328 png(file.path(fig_dir, "forest_study_level.png"), width = 1400, height = 1300, res = 150)

0329 forest(res_study, slab = paste0(study_level$author, " ", study_level$year),

0330 xlab = "Hedges' g (positive = priming better)",

0331 main = "Study-level forest plot (cross-check; three-level model in text)")

0332 dev.off()

0333

0334 png(file.path(fig_dir, "funnel_study_level.png"), width = 1000, height = 900, res = 150)

0335 funnel(res_study, main = "Funnel plot: study-level effects")

0336 dev.off()

0337

0338 png(file.path(fig_dir, "forest_effect_level.png"), width = 1500, height = 1800, res = 150)

0339 ord <- order(dat_es$year, dat_es$author, dat_es$outcome_domain_formal)

0340 forest(dat_es$yi[ord], dat_es$vi[ord],

0341 slab = paste0(dat_es$author[ord], " ", dat_es$year[ord], " - ", dat_es$outcome_name[ord]),

0342 xlab = "Hedges' g (positive = priming better)",

0343 main = "Effect-level estimates")

0344 dev.off()

0345

0346 plot_subgroups <- function(df, variable, filename, title) {

0347 d <- df %>% filter(subgroup_variable == variable) %>%

0348 mutate(subgroup = fct_reorder(subgroup, g))

0349 p <- ggplot(d, aes(x = g, y = subgroup)) +

0350 geom_vline(xintercept = 0, linetype = "dashed", color = "grey45") +

0351 geom_vline(xintercept = as.numeric(coef(res_main)), linetype = "dotted", color = "#8b1e1e") +

0352 geom_errorbarh(aes(xmin = ci_lb, xmax = ci_ub), height = 0.18, linewidth = 0.75, color = "#4f5d75") +

0353 geom_point(size = 2.8, color = "#2f6f9f") +

0354 labs(x = "Hedges' g (positive = priming better)", y = NULL, title = title,

0355 subtitle = "Dotted line = formal three-level overall estimate") +

0356 theme_minimal(base_size = 12) +

0357 theme(panel.grid.major.y = element_blank())

0358 ggsave(file.path(fig_dir, filename), p, width = 8, height = max(4, 0.45 * nrow(d) + 2), dpi = 300)

0359 }

0360 plot_subgroups(subgroups, "outcome_domain_formal", "subgroup_outcome_domain.png", "Subgroup analysis by outcome domain")

0361 plot_subgroups(subgroups, "priming_group", "subgroup_priming_type.png", "Subgroup analysis by priming type")

0362 plot_subgroups(subgroups, "interval_group", "subgroup_interval.png", "Subgroup analysis by priming-test interval")

0363

0364 loo <- map_dfr(unique(dat_es$study_id), function(s) {

0365 summarise_model(paste0("omit_", s), filter(dat_es, study_id != s)) %>%

0366 mutate(omitted = s)

0367 })

0368 write_csv(loo, file.path(tab_dir, "leave_one_study_out.csv"))

0369 p_loo <- ggplot(loo, aes(x = g, y = fct_reorder(omitted, g))) +

0370 geom_vline(xintercept = as.numeric(coef(res_main)), linetype = "dotted", color = "#8b1e1e") +

0371 geom_errorbarh(aes(xmin = ci_lb, xmax = ci_ub), height = 0.16, color = "#4f5d75") +

0372 geom_point(size = 2.3, color = "#2f6f9f") +

0373 labs(x = "Hedges' g after omitting study", y = NULL, title = "Leave-one-study-out analysis") +

0374 theme_minimal(base_size = 12) +

0375 theme(panel.grid.major.y = element_blank())

0376 ggsave(file.path(fig_dir, "leave_one_study_out.png"), p_loo, width = 8, height = 7, dpi = 300)

0377

0378 egger <- regtest(res_study, model = "rma")

0379 sink(file.path(log_dir, "publication_bias_tests.txt"))

0380 cat("Egger/regression test on study-level aggregated effects\n\n")

0381 print(egger)

0382 sink()

0383

0384 figure_metadata <- tibble(

0385 figure_file = c(

0386 "forest_study_level.png", "forest_effect_level.png", "funnel_study_level.png",

0387 "subgroup_outcome_domain.png", "subgroup_priming_type.png",

0388 "subgroup_interval.png", "leave_one_study_out.png"

0389 ),

0390 generated_by = "formal_meta_analysis.R",

0391 data_source = "data/extracted_data_master.csv",

0392 effect_size_metric = "Hedges' g; positive values favor priming",

0393 model = c(

0394 "Study-level random-effects REML with Hartung-Knapp cross-check",

0395 "Effect-level Hedges' g values",

0396 "Study-level random-effects REML",

0397 "Three-level REML subgroup summaries",

0398 "Three-level REML subgroup summaries",

0399 "Three-level REML subgroup summaries",

0400 "Three-level REML leave-one-study-out"

0401 ),

0402 dpi = c(150, 150, 150, 300, 300, 300, 300),

0403 created = analysis_date

0404 )

0405 write_csv(figure_metadata, file.path(meta_dir, "figure_metadata.csv"))

0406

0407 excluded_quant <- tribble(

0408 ~study, ~year, ~review_status, ~quantitative_reason,

0409 "Saez de Villarreal", 2007, "qualitative_only", "Percent change only; no usable SD or change SD for SMD.",

0410 "McGowan", 2017, "qualitative_only", "Primary time-trial outcome reported as percent change/CI; not converted for main SMD.",

0411 "Harrison", 2021, "qualitative_only", "Percent baseline/CI style reporting without raw mean/SD.",

0412 "Rud", 2021, "qualitative_only", "Percent difference +/- 95% CI only; no raw mean/SD.",

0413 "Holmberg", 2026, "qualitative_only", "LMM marginal means and MD 95% CI; no raw SD for SMD.",

0414 "Harrison", 2024, "qualitative_only", "Percent of control plus Cliff's delta; no raw mean/SD.",

0415 "Harrison", 2023, "qualitative_only", "Percent of control plus Cliff's delta; no raw mean/SD.",

0416 "González-García caffeine", 2023, "qualitative_only", "Percent of baseline plus Cohen's d; no raw mean/SD per condition."

0417 )

0418 write_csv(excluded_quant, file.path(tab_dir, "qualitative_only_not_quantitative.csv"))

0419

0420 cat("Formal analysis complete\n")

0421

sensitivity_gg2023.R

0001 # Sensitivity analysis for González-García 2023

0002 # Analysis 1: Exclude González-García 2023 entirely

0003 # Analysis 2: Use CI-derived SD instead of SWC-derived SD

0004

0005 library(metafor)

0006 library(clubSandwich)

0007 library(readr)

0008 library(dplyr)

0009

0010 root <- "D:/Priming_Meta_Analysis/Priming_Meta_Analysis/formal_extraction"

0011 data_path <- file.path(root, "data", "extracted_data_master.csv")

0012 out_dir <- file.path(root, "analysis", "output", "tables")

0013

0014 # --------------------------------------------------

0015 # Load and prep data (same as main script)

0016 # --------------------------------------------------

0017 raw <- read_csv(data_path, show_col_types = FALSE) %>%

0018 filter(include_main == 1, direction %in% c("higher_better", "lower_better")) %>%

0019 mutate(

0020 study_id = as.character(study_id),

0021 effect_id = as.character(effect_id),

0022 design = as.character(design),

0023 extraction_status = as.character(extraction_status),

0024 outcome_domain = as.character(outcome_domain),

0025 priming_type = as.character(priming_type),

0026 control_type = as.character(control_type),

0027 sd_priming = as.numeric(sd_priming),

0028 sd_control = as.numeric(sd_control),

0029 mean_priming = as.numeric(mean_priming),

0030 mean_control = as.numeric(mean_control),

0031 n_priming = as.numeric(n_priming),

0032 n_control = as.numeric(n_control),

0033 yi_reported = as.numeric(yi_reported),

0034 vi_reported = as.numeric(vi_reported)

0035 )

0036

0037 calc_cross_es <- function(df, r = 0.5) {

0038 df %>%

0039 mutate(

0040 sd_within = sqrt(sd_priming^2 + sd_control^2 - 2 * r * sd_priming * sd_control),

0041 d_rm = (mean_priming - mean_control) / sd_within,

0042 J = 1 - (3 / (4 * n_priming - 5)),

0043 yi_calc = J * d_rm,

0044 vi_calc = (1 / n_priming + yi_calc^2 / (2 * n_priming)) * J^2,

0045 es_method = "crossover rm-SMD; assumed r=0.5"

0046 )

0047 }

0048

0049 calc_prepost_es <- function(df, r = 0.5) {

0050 df %>%

0051 mutate(

0052 d_change = (mean_priming - mean_control) / sd_control,

0053 J = 1 - (3 / (4 * n_priming - 5)),

0054 yi_calc = J * d_change,

0055 vi_calc = (2 * (1 - r) / n_priming + yi_calc^2 / (2 * n_priming)) * J^2,

0056 es_method = "pre-post SMD; assumed r=0.5"

0057 )

0058 }

0059

0060 calc_ind_es <- function(df) {

0061 esc <- escalc(

0062 measure = "SMD",

0063 m1i = mean_priming, sd1i = sd_priming, n1i = n_priming,

0064 m2i = mean_control, sd2i = sd_control, n2i = n_control,

0065 data = df

0066 )

0067 df$yi_calc <- esc$yi

0068 df$vi_calc <- esc$vi

0069 df$es_method <- "independent-groups SMD"

0070 df

0071 }

0072

0073 compute_es <- function(df) {

0074 dat <- df %>% mutate(yi = NA_real_, vi = NA_real_, es_method = NA_character_)

0075

0076 reported_idx <- which(!is.na(dat$yi_reported) & !is.na(dat$vi_reported))

0077 if (length(reported_idx) > 0) {

0078 dat$yi[reported_idx] <- dat$yi_reported[reported_idx]

0079 dat$vi[reported_idx] <- dat$vi_reported[reported_idx]

0080 dat$es_method[reported_idx] <- "reported ES; approximate variance"

0081 }

0082

0083 remaining <- which(is.na(dat$yi))

0084 if (length(remaining) > 0) {

0085 pp_idx <- remaining[grepl("pre-post|repeated measures", dat$design[remaining], ignore.case = TRUE)]

0086 if (length(pp_idx) > 0) {

0087 tmp <- calc_prepost_es(dat[pp_idx, ], r = 0.5)

0088 dat[pp_idx, c("yi", "vi", "es_method")] <- tmp[, c("yi_calc", "vi_calc", "es_method")]

0089 }

0090 remaining <- which(is.na(dat$yi))

0091 cross_idx <- remaining[dat$design[remaining] == "crossover"]

0092 if (length(cross_idx) > 0) {

0093 tmp <- calc_cross_es(dat[cross_idx, ], r = 0.5)

0094 dat[cross_idx, c("yi", "vi", "es_method")] <- tmp[, c("yi_calc", "vi_calc", "es_method")]

0095 }

0096 remaining <- which(is.na(dat$yi))

0097 if (length(remaining) > 0) {

0098 tmp <- calc_ind_es(dat[remaining, ])

0099 dat[remaining, c("yi", "vi", "es_method")] <- tmp[, c("yi_calc", "vi_calc", "es_method")]

0100 }

0101 }

0102

0103 dat <- dat %>%

0104 mutate(

0105 yi = if_else(direction == "lower_better" & is.na(yi_reported), -yi, yi),

0106 vi = as.numeric(vi),

0107 se = sqrt(vi),

0108 ci_lb = yi - 1.96 * se,

0109 ci_ub = yi + 1.96 * se

0110 ) %>%

0111 filter(!is.na(yi), !is.na(vi), vi > 0)

0112

0113 dat

0114 }

0115

0116 fit_3l <- function(df, mods = NULL) {

0117 if (is.null(mods)) {

0118 rma.mv(yi = yi, V = vi, random = ~ 1 | study_id / effect_id,

0119 method = "REML", test = "t", data = df)

0120 } else {

0121 rma.mv(yi = yi, V = vi, mods = mods, random = ~ 1 | study_id / effect_id,

0122 method = "REML", test = "t", data = df)

0123 }

0124 }

0125

0126 calc_i2 <- function(res, df) {

0127 W <- diag(1 / df$vi)

0128 X <- model.matrix(res)

0129 P <- W - W %*% X %*% solve(t(X) %*% W %*% X) %*% t(X) %*% W

0130 typical_vi <- (nrow(df) - ncol(X)) / sum(diag(P))

0131 total_tau2 <- sum(res$sigma2)

0132 tibble(

0133 typical_vi = typical_vi,

0134 I2_total = 100 * total_tau2 / (total_tau2 + typical_vi),

0135 I2_level3 = 100 * res$sigma2[1] / (total_tau2 + typical_vi),

0136 I2_level2 = 100 * res$sigma2[2] / (total_tau2 + typical_vi)

0137 )

0138 }

0139

0140 summarise_model <- function(label, df) {

0141 if (n_distinct(df$study_id) < 2 || nrow(df) < 2) return(NULL)

0142 res <- fit_3l(df)

0143 pred <- predict(res)

0144 robust <- tryCatch(

0145 coef_test(res, vcov = "CR2", cluster = df$study_id, test = "Satterthwaite"),

0146 error = function(e) tibble(SE = NA_real_, p_Satt = NA_real_)

0147 )

0148 i2s <- calc_i2(res, df)

0149 tibble(

0150 analysis = label,

0151 k_studies = n_distinct(df$study_id),

0152 m_effects = nrow(df),

0153 g = as.numeric(coef(res)),

0154 ci_lb = res$ci.lb,

0155 ci_ub = res$ci.ub,

0156 p = res$pval,

0157 pi_lb = pred$cr.lb,

0158 pi_ub = pred$cr.ub,

0159 tau2_level3 = res$sigma2[1],

0160 tau2_level2 = res$sigma2[2],

0161 I2_total = i2s$I2_total,

0162 I2_level3 = i2s$I2_level3,

0163 I2_level2 = i2s$I2_level2,

0164 Q = res$QE,

0165 Q_p = res$QEp,

0166 robust_se = robust$SE[1],

0167 robust_p = robust$p_Satt[1]

0168 )

0169 }

0170

0171 # ==================================================

0172 # BASELINE: Current main model (for comparison)

0173 # ==================================================

0174 dat_base <- compute_es(raw)

0175 res_base <- summarise_model("BASELINE (current)", dat_base)

0176 cat("\n========== BASELINE (current k=18, m=46) ==========\n")

0177 print(res_base)

0178

0179 # ==================================================

0180 # ANALYSIS 1: Exclude González-García 2023

0181 # ==================================================

0182 raw_excl <- raw %>% filter(!grepl("Gonzalez-Garcia_2023", study_id))

0183 dat_excl <- compute_es(raw_excl)

0184 res_excl <- summarise_model("Exclude González-García 2023", dat_excl)

0185 cat("\n========== ANALYSIS 1: Exclude González-García 2023 ==========\n")

0186 print(res_excl)

0187

0188 # Show what changed

0189 excl_detail <- dat_excl %>%

0190 group_by(study_id) %>%

0191 summarise(yi_bar = mean(yi), n = n(), .groups = "drop") %>%

0192 arrange(desc(yi_bar))

0193 cat("\n--- Study-level effects after exclusion ---\n")

0194 print(excl_detail)

0195

0196 # ==================================================

0197 # ANALYSIS 2: Use CI-derived SD for González-García 2023

0198 # ==================================================

0199 # From Table 3 in paper:

0200 # Priming 6h: Mean=36.07, 95%CI=[30.96, 41.18], n=14

0201 # CI half-width = 5.11, t(13, 0.025)=2.160

0202 # SD = 5.11 * sqrt(14) / 2.160 = 8.85

0203 # Control 6h: Mean=36.21, 95%CI=[31.25, 41.18], n=14

0204 # CI half-width = 4.965

0205 # SD = 4.965 * sqrt(14) / 2.160 = 8.60

0206

0207 raw_ci <- raw %>%

0208 mutate(

0209 sd_priming = if_else(grepl("Gonzalez-Garcia_2023", study_id), 8.85, sd_priming),

0210 sd_control = if_else(grepl("Gonzalez-Garcia_2023", study_id), 8.60, sd_control)

0211 )

0212

0213 dat_ci <- compute_es(raw_ci)

0214 res_ci <- summarise_model("GG2023 with CI-derived SD", dat_ci)

0215 cat("\n========== ANALYSIS 2: GG2023 with CI-derived SD ==========\n")

0216 print(res_ci)

0217

0218 # Show the specific effect for González-García 2023 in both versions

0219 gg_base <- dat_base %>% filter(grepl("Gonzalez-Garcia_2023", study_id)) %>%

0220 select(study_id, effect_id, yi, vi, es_method)

0221 gg_ci <- dat_ci %>% filter(grepl("Gonzalez-Garcia_2023", study_id)) %>%

0222 select(study_id, effect_id, yi, vi, es_method)

0223

0224 cat("\n--- González-García 2023: BASELINE ---\n")

0225 print(gg_base)

0226 cat("\n--- González-García 2023: CI-derived SD ---\n")

0227 print(gg_ci)

0228

0229 # ==================================================

0230 # Save all results

0231 # ==================================================

0232 all_results <- bind_rows(res_base, res_excl, res_ci)

0233 write_csv(all_results, file.path(out_dir, "sensitivity_gg2023_results.csv"))

0234

0235 cat("\n========== COMPARISON TABLE ==========\n")

0236 print(all_results %>% select(analysis, k_studies, m_effects, g, ci_lb, ci_ub, p, robust_p, pi_lb, pi_ub))

0237

Figure and table generation code

00_formal_meta_analysis_all_figures.R

0001 # Formal meta-analysis for delayed exercise-based priming

0002 # Uses metafor for multilevel models and meta for study-level cross-checks.

0003

0004 options(repos = c(CRAN = "https://cran.rstudio.com/"))

0005 packages <- c(

0006 "metafor", "meta", "clubSandwich", "readr", "dplyr", "tidyr",

0007 "stringr", "ggplot2", "forcats", "purrr", "tibble"

0008 )

0009 missing <- packages[!(packages %in% rownames(installed.packages()))]

0010 if (length(missing) > 0) install.packages(missing, dependencies = TRUE)

0011

0012 library(metafor)

0013 library(meta)

0014 library(clubSandwich)

0015 library(readr)

0016 library(dplyr)

0017 library(tidyr)

0018 library(stringr)

0019 library(ggplot2)

0020 library(forcats)

0021 library(purrr)

0022 library(tibble)

0023

0024 script_arg <- commandArgs(trailingOnly = FALSE)

0025 script_path <- normalizePath(sub("^--file=", "", script_arg[grepl("^--file=", script_arg)][1]), winslash = "/", mustWork = TRUE)

0026 root <- normalizePath(file.path(dirname(script_path), "..", ".."), winslash = "/", mustWork = TRUE)

0027 data_path <- file.path(root, "data", "extracted_data_master.csv")

0028 out_dir <- file.path(root, "analysis", "output")

0029 fig_dir <- file.path(out_dir, "figures")

0030 tab_dir <- file.path(out_dir, "tables")

0031 meta_dir <- file.path(out_dir, "metadata")

0032 log_dir <- file.path(out_dir, "logs")

0033 obj_dir <- file.path(out_dir, "r_objects")

0034 dir.create(fig_dir, showWarnings = FALSE, recursive = TRUE)

0035 dir.create(tab_dir, showWarnings = FALSE, recursive = TRUE)

0036 dir.create(meta_dir, showWarnings = FALSE, recursive = TRUE)

0037 dir.create(log_dir, showWarnings = FALSE, recursive = TRUE)

0038 dir.create(obj_dir, showWarnings = FALSE, recursive = TRUE)

0039

0040 analysis_date <- format(Sys.time(), "%Y-%m-%d %H:%M:%S")

0041

0042 raw <- read_csv(data_path, show_col_types = FALSE) %>%

0043 filter(include_main == 1, direction %in% c("higher_better", "lower_better")) %>%

0044 mutate(

0045 study_id = as.character(study_id),

0046 effect_id = as.character(effect_id),

0047 design = as.character(design),

0048 outcome_domain = as.character(outcome_domain),

0049 priming_type = as.character(priming_type),

0050 control_type = as.character(control_type),

0051 outcome_domain_formal = case_when(

0052 outcome_domain %in% c("jump_power", "strength_power", "strength_force") ~ "strength_power",

0053 outcome_domain %in% c("sprint", "RSA", "COD", "speed_agility") ~ "speed_agility",

0054 outcome_domain %in% c("sport_specific") ~ "sport_specific",

0055 TRUE ~ outcome_domain

0056 ),

0057 priming_group = case_when(

0058 priming_type %in% c("sprint", "resisted_sprint") ~ "sprint_based",

0059 priming_type %in% c("ballistic") ~ "ballistic_jump",

0060 TRUE ~ priming_type

0061 ),

0062 interval_group = case_when(

0063 interval_h < 4 ~ "1_to_3h",

0064 interval_h < 6 ~ "4_to_5h",

0065 TRUE ~ "6h_or_more"

0066 )

0067 )

0068

0069 calc_cross_es <- function(df, r = 0.5) {

0070 df %>%

0071 mutate(

0072 sd_within = sqrt(sd_priming^2 + sd_control^2 - 2 * r * sd_priming * sd_control),

0073 d_rm = (mean_priming - mean_control) / sd_within,

0074 J = 1 - (3 / (4 * n_priming - 5)),

0075 yi_calc = J * d_rm,

0076 vi_calc = (1 / n_priming + yi_calc^2 / (2 * n_priming)) * J^2,

0077 es_method = "crossover rm-SMD; assumed r=0.5"

0078 )

0079 }

0080

0081 calc_prepost_es <- function(df, r = 0.5) {

0082 df %>%

0083 mutate(

0084 d_change = (mean_priming - mean_control) / sd_control,

0085 J = 1 - (3 / (4 * n_priming - 5)),

0086 yi_calc = J * d_change,

0087 vi_calc = (2 * (1 - r) / n_priming + yi_calc^2 / (2 * n_priming)) * J^2,

0088 es_method = "pre-post SMD; assumed r=0.5"

0089 )

0090 }

0091

0092 calc_ind_es <- function(df) {

0093 esc <- escalc(

0094 measure = "SMD",

0095 m1i = mean_priming, sd1i = sd_priming, n1i = n_priming,

0096 m2i = mean_control, sd2i = sd_control, n2i = n_control,

0097 data = df

0098 )

0099 df$yi_calc <- esc$yi

0100 df$vi_calc <- esc$vi

0101 df$es_method <- "independent-groups SMD"

0102 df

0103 }

0104

0105 dat_es <- raw %>% mutate(yi = NA_real_, vi = NA_real_, es_method = NA_character_)

0106

0107 reported_idx <- which(!is.na(dat_es$yi_reported) & !is.na(dat_es$vi_reported))

0108 if (length(reported_idx) > 0) {

0109 dat_es$yi[reported_idx] <- dat_es$yi_reported[reported_idx]

0110 dat_es$vi[reported_idx] <- dat_es$vi_reported[reported_idx]

0111 dat_es$es_method[reported_idx] <- "reported ES; approximate variance"

0112 }

0113

0114 remaining <- which(is.na(dat_es$yi))

0115 if (length(remaining) > 0) {

0116 pp_idx <- remaining[grepl("pre-post|repeated measures", dat_es$design[remaining], ignore.case = TRUE)]

0117 if (length(pp_idx) > 0) {

0118 tmp <- calc_prepost_es(dat_es[pp_idx, ], r = 0.5)

0119 dat_es[pp_idx, c("yi", "vi", "es_method")] <- tmp[, c("yi_calc", "vi_calc", "es_method")]

0120 }

0121 remaining <- which(is.na(dat_es$yi))

0122 cross_idx <- remaining[dat_es$design[remaining] == "crossover"]

0123 if (length(cross_idx) > 0) {

0124 tmp <- calc_cross_es(dat_es[cross_idx, ], r = 0.5)

0125 dat_es[cross_idx, c("yi", "vi", "es_method")] <- tmp[, c("yi_calc", "vi_calc", "es_method")]

0126 }

0127 remaining <- which(is.na(dat_es$yi))

0128 if (length(remaining) > 0) {

0129 tmp <- calc_ind_es(dat_es[remaining, ])

0130 dat_es[remaining, c("yi", "vi", "es_method")] <- tmp[, c("yi_calc", "vi_calc", "es_method")]

0131 }

0132 }

0133

0134 dat_es <- dat_es %>%

0135 mutate(

0136 yi = if_else(direction == "lower_better" & is.na(yi_reported), -yi, yi),

0137 vi = as.numeric(vi),

0138 se = sqrt(vi),

0139 ci_lb = yi - 1.96 * se,

0140 ci_ub = yi + 1.96 * se

0141 ) %>%

0142 filter(!is.na(yi), !is.na(vi), vi > 0)

0143

0144 write_csv(dat_es, file.path(tab_dir, "formal_effect_sizes.csv"))

0145

0146 fit_3l <- function(df, mods = NULL) {

0147 if (is.null(mods)) {

0148 rma.mv(yi = yi, V = vi, random = ~ 1 | study_id / effect_id,

0149 method = "REML", test = "t", data = df)

0150 } else {

0151 rma.mv(yi = yi, V = vi, mods = mods, random = ~ 1 | study_id / effect_id,

0152 method = "REML", test = "t", data = df)

0153 }

0154 }

0155

0156 calc_i2 <- function(res, df) {

0157 W <- diag(1 / df$vi)

0158 X <- model.matrix(res)

0159 P <- W - W %*% X %*% solve(t(X) %*% W %*% X) %*% t(X) %*% W

0160 typical_vi <- (nrow(df) - ncol(X)) / sum(diag(P))

0161 total_tau2 <- sum(res$sigma2)

0162 tibble(

0163 typical_vi = typical_vi,

0164 I2_total = 100 * total_tau2 / (total_tau2 + typical_vi),

0165 I2_level3 = 100 * res$sigma2[1] / (total_tau2 + typical_vi),

0166 I2_level2 = 100 * res$sigma2[2] / (total_tau2 + typical_vi)

0167 )

0168 }

0169

0170 summarise_model <- function(label, df) {

0171 if (n_distinct(df$study_id) < 2 || nrow(df) < 2) return(NULL)

0172 res <- fit_3l(df)

0173 pred <- predict(res)

0174 robust <- tryCatch(

0175 coef_test(res, vcov = "CR2", cluster = df$study_id, test = "Satterthwaite"),

0176 error = function(e) tibble(SE = NA_real_, p_Satt = NA_real_)

0177 )

0178 i2s <- calc_i2(res, df)

0179 tibble(

0180 analysis = label,

0181 k_studies = n_distinct(df$study_id),

0182 m_effects = nrow(df),

0183 g = as.numeric(coef(res)),

0184 ci_lb = res$ci.lb,

0185 ci_ub = res$ci.ub,

0186 p = res$pval,

0187 pi_lb = pred$cr.lb,

0188 pi_ub = pred$cr.ub,

0189 tau2_level3 = res$sigma2[1],

0190 tau2_level2 = res$sigma2[2],

0191 I2_total = i2s$I2_total,

0192 I2_level3 = i2s$I2_level3,

0193 I2_level2 = i2s$I2_level2,

0194 Q = res$QE,

0195 Q_p = res$QEp,

0196 robust_se = robust$SE[1],

0197 robust_p = robust$p_Satt[1]

0198 )

0199 }

0200

0201 res_main <- fit_3l(dat_es)

0202 robust_main <- coef_test(res_main, vcov = "CR2", cluster = dat_es$study_id, test = "Satterthwaite")

0203 pred_main <- predict(res_main)

0204

0205 preferred_domain_order <- c(

0206 "sport_specific" = 1,

0207 "speed_agility" = 2,

0208 "strength_power" = 3

0209 )

0210 dat_one <- dat_es %>%

0211 mutate(priority = if_else(outcome_domain_formal %in% names(preferred_domain_order),

0212 preferred_domain_order[outcome_domain_formal], 99)) %>%

0213 arrange(study_id, priority, effect_id) %>%

0214 group_by(study_id) %>%

0215 slice(1) %>%

0216 ungroup()

0217

0218 models <- bind_rows(

0219 summarise_model("Main formal model", dat_es),

0220 summarise_model("Crossover only", filter(dat_es, design == "crossover")),

0221 summarise_model(">=4 h only", filter(dat_es, interval_h >= 4)),

0222 summarise_model("≥6 h only", filter(dat_es, interval_h >= 6)),

0223 summarise_model("One preferred outcome per study", dat_one)

0224 )

0225 write_csv(models, file.path(tab_dir, "overall_and_sensitivity_models.csv"))

0226

0227 subgroup_model <- function(var) {

0228 dat_es %>%

0229 filter(!is.na(.data[[var]])) %>%

0230 group_split(.data[[var]]) %>%

0231 map_dfr(function(df) {

0232 label <- as.character(df[[var]][1])

0233 out <- summarise_model(label, df)

0234 if (is.null(out)) {

0235 w <- 1 / df$vi

0236 tibble(

0237 analysis = label, k_studies = n_distinct(df$study_id), m_effects = nrow(df),

0238 g = sum(w * df$yi) / sum(w),

0239 ci_lb = g - 1.96 * sqrt(1 / sum(w)),

0240 ci_ub = g + 1.96 * sqrt(1 / sum(w)),

0241 p = NA_real_, pi_lb = NA_real_, pi_ub = NA_real_,

0242 tau2_level3 = NA_real_, tau2_level2 = NA_real_,

0243 I2_total = NA_real_, I2_level3 = NA_real_, I2_level2 = NA_real_,

0244 Q = NA_real_, Q_p = NA_real_, robust_se = NA_real_, robust_p = NA_real_

0245 )

0246 } else out

0247 }) %>%

0248 rename(subgroup = analysis) %>%

0249 mutate(subgroup_variable = var, .before = 1)

0250 }

0251

0252 subgroups <- bind_rows(

0253 subgroup_model("outcome_domain_formal"),

0254 subgroup_model("priming_group"),

0255 subgroup_model("interval_group"),

0256 subgroup_model("control_type"),

0257 subgroup_model("athlete_level")

0258 )

0259 write_csv(subgroups, file.path(tab_dir, "subgroup_models.csv"))

0260

0261 moderator_tests <- list(

0262 outcome_domain_formal = ~ factor(outcome_domain_formal),

0263 priming_group = ~ factor(priming_group),

0264 interval_group = ~ factor(interval_group),

0265 control_type = ~ factor(control_type),

0266 athlete_level = ~ factor(athlete_level)

0267 ) %>%

0268 imap_dfr(function(mod, name) {

0269 fit <- tryCatch(fit_3l(dat_es, mods = mod), error = function(e) NULL)

0270 if (is.null(fit)) return(tibble(moderator = name, QM = NA_real_, df = NA_real_, p = NA_real_))

0271 tibble(moderator = name, QM = fit$QM, df = fit$m - 1, p = fit$QMp)

0272 })

0273 write_csv(moderator_tests, file.path(tab_dir, "moderator_tests.csv"))

0274

0275 study_level <- dat_es %>%

0276 group_by(study_id, author, year) %>%

0277 summarise(

0278 yi = weighted.mean(yi, w = 1 / vi),

0279 vi = 1 / sum(1 / vi),

0280 m_effects = n(),

0281 .groups = "drop"

0282 ) %>%

0283 arrange(year, author)

0284 write_csv(study_level, file.path(tab_dir, "study_level_aggregated_effects.csv"))

0285

0286 res_study <- rma(yi, vi, data = study_level, method = "REML", test = "knha")

0287 meta_study <- metagen(

0288 TE = yi, seTE = sqrt(vi), studlab = study_id,

0289 data = study_level, sm = "SMD",

0290 common = FALSE, random = TRUE, method.tau = "REML",

0291 method.random.ci = "HK"

0292 )

0293

0294 sink(file.path(log_dir, "formal_model_output.txt"))

0295 cat("Formal delayed exercise-based priming meta-analysis\n")

0296 cat("Analysis date:", analysis_date, "\n\n")

0297 cat("Studies:", n_distinct(dat_es$study_id), "\n")

0298 cat("Effect sizes:", nrow(dat_es), "\n\n")

0299 cat("metafor three-level model\n")

0300 print(summary(res_main))

0301 cat("\nPrediction interval\n")

0302 print(pred_main)

0303 cat("\nCR2 robust test\n")

0304 print(robust_main)

0305 cat("\nmeta package study-level cross-check\n")

0306 print(summary(meta_study))

0307 cat("\nSensitivity models\n")

0308 print(models)

0309 cat("\nModerator tests\n")

0310 print(moderator_tests)

0311 sink()

0312

0313 saveRDS(list(

0314 dat_es = dat_es, res_main = res_main, robust_main = robust_main,

0315 pred_main = pred_main, study_level = study_level, res_study = res_study,

0316 meta_study = meta_study, models = models, subgroups = subgroups,

0317 moderator_tests = moderator_tests

0318 ), file.path(obj_dir, "formal_meta_objects.rds"))

0319

0320 write_csv(tibble(

0321 item = c("analysis_date", "k_studies", "m_effects", "main_g", "main_ci_lb", "main_ci_ub",

0322 "main_p", "robust_p", "prediction_lb", "prediction_ub"),

0323 value = c(analysis_date, n_distinct(dat_es$study_id), nrow(dat_es),

0324 as.numeric(coef(res_main)), res_main$ci.lb, res_main$ci.ub, res_main$pval,

0325 robust_main$p_Satt[1], pred_main$cr.lb, pred_main$cr.ub)

0326 ), file.path(tab_dir, "formal_key_results.csv"))

0327

0328 png(file.path(fig_dir, "forest_study_level.png"), width = 1400, height = 1300, res = 150)

0329 forest(res_study, slab = paste0(study_level$author, " ", study_level$year),

0330 xlab = "Hedges' g (positive = priming better)",

0331 main = "Delayed exercise-based priming: study-level random-effects model")

0332 dev.off()

0333

0334 png(file.path(fig_dir, "funnel_study_level.png"), width = 1000, height = 900, res = 150)

0335 funnel(res_study, main = "Funnel plot: study-level effects")

0336 dev.off()

0337

0338 png(file.path(fig_dir, "forest_effect_level.png"), width = 1500, height = 1800, res = 150)

0339 ord <- order(dat_es$year, dat_es$author, dat_es$outcome_domain_formal)

0340 forest(dat_es$yi[ord], dat_es$vi[ord],

0341 slab = paste0(dat_es$author[ord], " ", dat_es$year[ord], " - ", dat_es$outcome_name[ord]),

0342 xlab = "Hedges' g (positive = priming better)",

0343 main = "Effect-level estimates")

0344 dev.off()

0345

0346 plot_subgroups <- function(df, variable, filename, title) {

0347 d <- df %>% filter(subgroup_variable == variable) %>%

0348 mutate(subgroup = fct_reorder(subgroup, g))

0349 p <- ggplot(d, aes(x = g, y = subgroup)) +

0350 geom_vline(xintercept = 0, linetype = "dashed", color = "grey45") +

0351 geom_vline(xintercept = as.numeric(coef(res_main)), linetype = "dotted", color = "#8b1e1e") +

0352 geom_errorbarh(aes(xmin = ci_lb, xmax = ci_ub), height = 0.18, linewidth = 0.75, color = "#4f5d75") +

0353 geom_point(size = 2.8, color = "#2f6f9f") +

0354 labs(x = "Hedges' g (positive = priming better)", y = NULL, title = title,

0355 subtitle = "Dotted line = formal three-level overall estimate") +

0356 theme_minimal(base_size = 12) +

0357 theme(panel.grid.major.y = element_blank())

0358 ggsave(file.path(fig_dir, filename), p, width = 8, height = max(4, 0.45 * nrow(d) + 2), dpi = 300)

0359 }

0360 plot_subgroups(subgroups, "outcome_domain_formal", "subgroup_outcome_domain.png", "Subgroup analysis by outcome domain")

0361 plot_subgroups(subgroups, "priming_group", "subgroup_priming_type.png", "Subgroup analysis by priming type")

0362 plot_subgroups(subgroups, "interval_group", "subgroup_interval.png", "Subgroup analysis by priming-test interval")

0363

0364 loo <- map_dfr(unique(dat_es$study_id), function(s) {

0365 summarise_model(paste0("omit_", s), filter(dat_es, study_id != s)) %>%

0366 mutate(omitted = s)

0367 })

0368 write_csv(loo, file.path(tab_dir, "leave_one_study_out.csv"))

0369 p_loo <- ggplot(loo, aes(x = g, y = fct_reorder(omitted, g))) +

0370 geom_vline(xintercept = as.numeric(coef(res_main)), linetype = "dotted", color = "#8b1e1e") +

0371 geom_errorbarh(aes(xmin = ci_lb, xmax = ci_ub), height = 0.16, color = "#4f5d75") +

0372 geom_point(size = 2.3, color = "#2f6f9f") +

0373 labs(x = "Hedges' g after omitting study", y = NULL, title = "Leave-one-study-out analysis") +

0374 theme_minimal(base_size = 12) +

0375 theme(panel.grid.major.y = element_blank())

0376 ggsave(file.path(fig_dir, "leave_one_study_out.png"), p_loo, width = 8, height = 7, dpi = 300)

0377

0378 egger <- regtest(res_study, model = "rma")

0379 sink(file.path(log_dir, "publication_bias_tests.txt"))

0380 cat("Egger/regression test on study-level aggregated effects\n\n")

0381 print(egger)

0382 sink()

0383

0384 figure_metadata <- tibble(

0385 figure_file = c(

0386 "forest_study_level.png", "forest_effect_level.png", "funnel_study_level.png",

0387 "subgroup_outcome_domain.png", "subgroup_priming_type.png",

0388 "subgroup_interval.png", "leave_one_study_out.png"

0389 ),

0390 generated_by = "formal_meta_analysis.R",

0391 data_source = "data/extracted_data_master.csv",

0392 effect_size_metric = "Hedges' g; positive values favor priming",

0393 model = c(

0394 "Study-level random-effects REML with Hartung-Knapp cross-check",

0395 "Effect-level Hedges' g values",

0396 "Study-level random-effects REML",

0397 "Three-level REML subgroup summaries",

0398 "Three-level REML subgroup summaries",

0399 "Three-level REML subgroup summaries",

0400 "Three-level REML subgroup summaries",

0401 "Three-level REML leave-one-study-out"

0402 ),

0403 dpi = c(150, 150, 150, 300, 300, 300, 300, 300),

0404 created = analysis_date

0405 )

0406 write_csv(figure_metadata, file.path(meta_dir, "figure_metadata.csv"))

0407

0408 excluded_quant <- tribble(

0409 ~study, ~year, ~review_status, ~quantitative_reason,

0410 "Saez de Villarreal", 2007, "qualitative_only", "Percent change only; no usable SD or change SD for SMD.",

0411 "McGowan", 2017, "qualitative_only", "Primary time-trial outcome reported as percent change/CI; not converted for main SMD.",

0412 "Harrison", 2021, "qualitative_only", "Percent baseline/CI style reporting without raw mean/SD.",

0413 "Rud", 2021, "qualitative_only", "Percent difference +/- 95% CI only; no raw mean/SD.",

0414 "Holmberg", 2026, "qualitative_only", "LMM marginal means and MD 95% CI; no raw SD for SMD.",

0415 "Harrison", 2024, "qualitative_only", "Percent of control plus Cliff's delta; no raw mean/SD.",

0416 "Harrison", 2023, "qualitative_only", "Percent of control plus Cliff's delta; no raw mean/SD.",

0417 "González-García caffeine", 2023, "qualitative_only", "Percent of baseline plus Cohen's d; no raw mean/SD per condition."

0418 )

0419 write_csv(excluded_quant, file.path(tab_dir, "qualitative_only_not_quantitative.csv"))

0420

0421 cat("Formal analysis complete\n")

0422

01_generate_figures_from_exported_data.R

0001 # Rebuild all formal figures from exported figure_data CSV files.

0002 # This script is intentionally self-contained for figure reproducibility.

0003

0004 options(repos = c(CRAN = "https://cran.rstudio.com/"))

0005 packages <- c("metafor", "readr", "dplyr", "ggplot2", "forcats")

0006 missing <- packages[!(packages %in% rownames(installed.packages()))]

0007 if (length(missing) > 0) install.packages(missing, dependencies = TRUE)

0008

0009 library(metafor)

0010 library(readr)

0011 library(dplyr)

0012 library(ggplot2)

0013 library(forcats)

0014

0015 script_arg <- commandArgs(trailingOnly = FALSE)

0016 script_path <- normalizePath(sub("^--file=", "", script_arg[grepl("^--file=", script_arg)][1]), winslash = "/", mustWork = TRUE)

0017 root <- normalizePath(file.path(dirname(script_path), ".."), winslash = "/", mustWork = TRUE)

0018 data_dir <- file.path(root, "figure_data")

0019 fig_dir <- file.path(root, "figures")

0020 dir.create(fig_dir, showWarnings = FALSE, recursive = TRUE)

0021

0022 study <- read_csv(file.path(data_dir, "forest_study_level_data.csv"), show_col_types = FALSE)

0023 effect <- read_csv(file.path(data_dir, "forest_effect_level_data.csv"), show_col_types = FALSE)

0024 loo <- read_csv(file.path(data_dir, "leave_one_study_out_data.csv"), show_col_types = FALSE)

0025

0026 res_study <- rma(yi, vi, data = study, method = "REML", test = "knha")

0027

0028 png(file.path(fig_dir, "forest_study_level.png"), width = 1400, height = 1300, res = 150)

0029 forest(res_study, slab = paste0(study$author, " ", study$year),

0030 xlab = "Hedges' g (positive = priming better)",

0031 main = "Delayed exercise-based priming: study-level random-effects model")

0032 dev.off()

0033

0034 png(file.path(fig_dir, "funnel_study_level.png"), width = 1000, height = 900, res = 150)

0035 funnel(res_study, main = "Funnel plot: study-level effects")

0036 dev.off()

0037

0038 png(file.path(fig_dir, "forest_effect_level.png"), width = 1500, height = 1800, res = 150)

0039 ord <- order(effect$year, effect$author, effect$outcome_domain_formal)

0040 forest(effect$yi[ord], effect$vi[ord],

0041 slab = paste0(effect$author[ord], " ", effect$year[ord], " - ", effect$outcome_name[ord]),

0042 xlab = "Hedges' g (positive = priming better)",

0043 main = "Effect-level estimates")

0044 dev.off()

0045

0046 plot_subgroup_file <- function(csv_name, output_name, title) {

0047 d <- read_csv(file.path(data_dir, csv_name), show_col_types = FALSE) %>%

0048 mutate(subgroup = fct_reorder(subgroup, g))

0049 overall <- read_csv(file.path(root, "tables", "formal_key_results.csv"), show_col_types = FALSE) %>%

0050 filter(item == "main_g") %>%

0051 pull(value) %>%

0052 as.numeric()

0053 p <- ggplot(d, aes(x = g, y = subgroup)) +

0054 geom_vline(xintercept = 0, linetype = "dashed", color = "grey45") +

0055 geom_vline(xintercept = overall, linetype = "dotted", color = "#8b1e1e") +

0056 geom_errorbar(aes(xmin = ci_lb, xmax = ci_ub), width = 0.18,

0057 orientation = "y", linewidth = 0.75, color = "#4f5d75") +

0058 geom_point(size = 2.8, color = "#2f6f9f") +

0059 labs(x = "Hedges' g (positive = priming better)", y = NULL,

0060 title = title, subtitle = "Dotted line = formal three-level overall estimate") +

0061 theme_minimal(base_size = 12) +

0062 theme(panel.grid.major.y = element_blank())

0063 ggsave(file.path(fig_dir, output_name), p, width = 8, height = max(4, 0.45 * nrow(d) + 2), dpi = 300)

0064 }

0065

0066 plot_subgroup_file("subgroup_outcome_domain_data.csv", "subgroup_outcome_domain.png",

0067 "Subgroup analysis by outcome domain")

0068 plot_subgroup_file("subgroup_priming_type_data.csv", "subgroup_priming_type.png",

0069 "Subgroup analysis by priming type")

0070 plot_subgroup_file("subgroup_interval_data.csv", "subgroup_interval.png",

0071 "Subgroup analysis by priming-test interval")

0072

0073 overall <- read_csv(file.path(root, "tables", "formal_key_results.csv"), show_col_types = FALSE) %>%

0074 filter(item == "main_g") %>%

0075 pull(value) %>%

0076 as.numeric()

0077 p_loo <- ggplot(loo, aes(x = g, y = fct_reorder(omitted, g))) +

0078 geom_vline(xintercept = overall, linetype = "dotted", color = "#8b1e1e") +

0079 geom_errorbar(aes(xmin = ci_lb, xmax = ci_ub), width = 0.16,

0080 orientation = "y", color = "#4f5d75") +

0081 geom_point(size = 2.3, color = "#2f6f9f") +

0082 labs(x = "Hedges' g after omitting study", y = NULL,

0083 title = "Leave-one-study-out analysis") +

0084 theme_minimal(base_size = 12) +

0085 theme(panel.grid.major.y = element_blank())

0086 ggsave(file.path(fig_dir, "leave_one_study_out.png"), p_loo, width = 8, height = 7, dpi = 300)

0087

0088 cat("All figures regenerated from figure_data.\n")

0089

generate_fig1_from_local_data.R

0001 from pathlib import Path

0002

0003 from openpyxl import Workbook

0004 from PIL import Image, ImageDraw, ImageFont

0005

0006

0007 ROOT = Path(__file__).resolve().parents[3]

0008 FORMAL = Path(__file__).resolve().parents[2]

0009 DATA = FORMAL / "data"

0010 OUT_FIG = FORMAL / "formal_outputs" / "figures"

0011 OUT_DATA = FORMAL / "formal_outputs" / "figure_data"

0012 SUPP_FIG = FORMAL / "supplementary_materials" / "01_figures"

0013 SUPP_DATA = FORMAL / "supplementary_materials" / "04_data"

0014 SEARCH_FLOW = next((p / "PRISMA_flow" for p in ROOT.iterdir() if (p / "PRISMA_flow").exists()), ROOT / "PRISMA_flow")

0015

0016 for p in [OUT_FIG, OUT_DATA, SUPP_FIG, SUPP_DATA, SEARCH_FLOW]:

0017 p.mkdir(parents=True, exist_ok=True)

0018

0019 counts = [

0020 ("Records identified", 3095, "EndNote raw export before deduplication"),

0021 ("Duplicate records removed", 1107, "Deduplication by title/DOI/PMID"),

0022 ("Records screened after deduplication", 1988, "Title/abstract screening"),

0023 ("Records excluded at title/abstract", 1776, "Not delayed exercise-based priming / not eligible"),

0024 ("Reports assessed for eligibility", 212, "Full-text/web-assisted eligibility assessment"),

0025 ("Reports excluded at eligibility", 186, "Did not meet PICOS, publication type, comparator, intervention, or data criteria"),

0026 ("Studies included in qualitative synthesis", 26, "18 quantitative + 8 SMD-incompatible narrative-only"),

0027 ("Studies included in quantitative synthesis", 18, "46 effect sizes"),

0028 ]

0029

0030 csv_text = "Step,Count,Notes\n" + "\n".join(f'{step},{count},"{note}"' for step, count, note in counts) + "\n"

0031 for target in [DATA / "prisma_formal_counts.csv", SUPP_DATA / "prisma_formal_counts.csv", OUT_DATA / "prisma_formal_counts.csv"]:

0032 target.parent.mkdir(parents=True, exist_ok=True)

0033 target.write_text(csv_text, encoding="utf-8")

0034

0035 wb = Workbook()

0036 ws = wb.active

0037 ws.title = "PRISMA counts"

0038 ws.append(["Step", "Count", "Notes"])

0039 for row in counts:

0040 ws.append(list(row))

0041 for col, width in {"A": 42, "B": 12, "C": 78}.items():

0042 ws.column_dimensions[col].width = width

0043 for target in [DATA / "prisma_formal_counts.xlsx", SUPP_DATA / "prisma_formal_counts.xlsx", SEARCH_FLOW / "PRISMA_2020_tracking.xlsx"]:

0044 target.parent.mkdir(parents=True, exist_ok=True)

0045 wb.save(target)

0046

0047

0048 def font(size=32, bold=False):

0049 candidates = [r"C:\Windows\Fonts\arialbd.ttf" if bold else r"C:\Windows\Fonts\arial.ttf"]

0050 for candidate in candidates:

0051 if Path(candidate).exists():

0052 return ImageFont.truetype(candidate, size=size)

0053 return ImageFont.load_default()

0054

0055 W, H = 1800, 2350

0056 img = Image.new("RGB", (W, H), "white")

0057 d = ImageDraw.Draw(img)

0058 dark = "#1f2937"

0059 line = "#4b5563"

0060 fill = "#f8fafc"

0061 edge = "#111827"

0062

0063 d.text((W // 2, 70), "PRISMA 2020 Flow Diagram", anchor="mm", font=font(42, True), fill=dark)

0064 boxes = [

0065 ("id", 260, 150, 1120, 290, "Records identified from databases and other sources\n(n = 3,095)"),

0066 ("dup", 260, 410, 1120, 550, "Duplicate records removed\n(n = 1,107)"),

0067 ("screen", 260, 670, 1120, 810, "Records screened after deduplication\n(n = 1,988)"),

0068 ("ta_exc", 1240, 670, 1740, 810, "Records excluded at title/abstract\n(n = 1,776)"),

0069 ("full", 260, 1050, 1120, 1190, "Reports assessed for eligibility\n(n = 212)"),

0070 ("full_exc", 1240, 1028, 1740, 1258, "Reports excluded at eligibility\n(n = 186)\nPICOS/publication type,\nintervention, comparator,\nor data criteria"),

0071 ("qual", 260, 1450, 1120, 1590, "Studies included in qualitative synthesis\n(n = 26)"),

0072 ("quant", 260, 1770, 1120, 1910, "Studies included in quantitative synthesis\n(n = 18; 46 effect sizes)"),

0073 ("narr", 260, 2050, 1120, 2190, "Qualitative-only eligible studies\n(n = 8; not pooled for SMD)"),

0074 ]

0075 coords = {}

0076 for key, x1, y1, x2, y2, text in boxes:

0077 coords[key] = (x1, y1, x2, y2)

0078 d.rounded_rectangle((x1, y1, x2, y2), radius=18, fill=fill, outline=edge, width=3)

0079 lines = text.split("\n")

0080 y = (y1 + y2) / 2 - len(lines) * 38 / 2 + 18

0081 for line_text in lines:

0082 d.text(((x1 + x2) / 2, y), line_text, anchor="mm", font=font(30 if len(line_text) < 46 else 24), fill=dark)

0083 y += 38

0084

0085

0086 def down(a, b):

0087 x = (coords[a][0] + coords[a][2]) / 2

0088 y1 = coords[a][3]

0089 y2 = coords[b][1]

0090 d.line((x, y1, x, y2), fill=line, width=5)

0091 d.polygon([(x, y2), (x - 12, y2 - 22), (x + 12, y2 - 22)], fill=line)

0092

0093

0094 def right(a, b):

0095 x1 = coords[a][2]

0096 y = (coords[a][1] + coords[a][3]) / 2

0097 x2 = coords[b][0]

0098 d.line((x1, y, x2, y), fill=line, width=5)

0099 d.polygon([(x2, y), (x2 - 22, y - 12), (x2 - 22, y + 12)], fill=line)

0100

0101 for a, b in [("id", "dup"), ("dup", "screen"), ("screen", "full"), ("full", "qual"), ("qual", "quant"), ("quant", "narr")]:

0102 down(a, b)

0103 right("screen", "ta_exc")

0104 right("full", "full_exc")

0105

0106 d.text((900, 2285), "SMD = standardized mean difference.", anchor="mm", font=font(25), fill="#374151")

0107 for target in [OUT_FIG / "prisma_2020_flow_diagram.png", SUPP_FIG / "SF1_prisma_2020_flow_diagram.png", SEARCH_FLOW / "PRISMA_2020_flow_diagram.png"]:

0108 target.parent.mkdir(parents=True, exist_ok=True)

0109 img.save(target, dpi=(300, 300))

0110

0111 print(OUT_FIG / "prisma_2020_flow_diagram.png")

0112

02_export_figure_data_tables_manifest.R

0001 # Export figure source data, summary tables, an Excel workbook, and the output manifest

0002 # from the current formal analysis outputs.

0003

0004 options(repos = c(CRAN = "https://cran.rstudio.com/"))

0005 packages <- c("readr", "dplyr", "openxlsx", "tools", "tibble")

0006 missing <- packages[!(packages %in% rownames(installed.packages()))]

0007 if (length(missing) > 0) install.packages(missing, dependencies = TRUE)

0008

0009 library(readr)

0010 library(dplyr)

0011 library(openxlsx)

0012 library(tools)

0013

0014 script_arg <- commandArgs(trailingOnly = FALSE)

0015 script_path <- normalizePath(sub("^--file=", "", script_arg[grepl("^--file=", script_arg)][1]), winslash = "/", mustWork = TRUE)

0016 root <- normalizePath(file.path(dirname(script_path), ".."), winslash = "/", mustWork = TRUE)

0017 tables_dir <- file.path(root, "tables")

0018 figure_data_dir <- file.path(root, "figure_data")

0019 metadata_dir <- file.path(root, "metadata")

0020 dir.create(figure_data_dir, showWarnings = FALSE, recursive = TRUE)

0021 dir.create(metadata_dir, showWarnings = FALSE, recursive = TRUE)

0022

0023 effect <- read_csv(file.path(tables_dir, "formal_effect_sizes.csv"), show_col_types = FALSE)

0024 study <- read_csv(file.path(tables_dir, "study_level_aggregated_effects.csv"), show_col_types = FALSE)

0025 loo <- read_csv(file.path(tables_dir, "leave_one_study_out.csv"), show_col_types = FALSE)

0026 subgroups <- read_csv(file.path(tables_dir, "subgroup_models.csv"), show_col_types = FALSE)

0027

0028 write_csv(effect, file.path(figure_data_dir, "forest_effect_level_data.csv"))

0029 write_csv(study, file.path(figure_data_dir, "forest_study_level_data.csv"))

0030 write_csv(study, file.path(figure_data_dir, "funnel_study_level_data.csv"))

0031 write_csv(loo, file.path(figure_data_dir, "leave_one_study_out_data.csv"))

0032

0033 subgroup_exports <- list(

0034 outcome_domain_formal = "subgroup_outcome_domain_data.csv",

0035 priming_group = "subgroup_priming_type_data.csv",

0036 interval_group = "subgroup_interval_data.csv",

0037 )

0038 for (moderator_name in names(subgroup_exports)) {

0039 subgroups %>%

0040 filter(subgroup_variable == moderator_name) %>%

0041 write_csv(file.path(figure_data_dir, subgroup_exports[[moderator_name]]))

0042 }

0043

0044 study_characteristics <- effect %>%

0045 group_by(study_id, author, year, sport, athlete_level, design, priming_group,

0046 control_type, interval_group, title, journal, doi_pmid) %>%

0047 summarise(

0048 n_effects = n(),

0049 outcome_domains = paste(sort(unique(outcome_domain_formal)), collapse = "; "),

0050 min_interval_h = suppressWarnings(min(interval_h, na.rm = TRUE)),

0051 max_interval_h = suppressWarnings(max(interval_h, na.rm = TRUE)),

0052 .groups = "drop"

0053 ) %>%

0054 arrange(year, author)

0055 study_characteristics$min_interval_h[is.infinite(study_characteristics$min_interval_h)] <- NA_real_

0056 study_characteristics$max_interval_h[is.infinite(study_characteristics$max_interval_h)] <- NA_real_

0057 write_csv(study_characteristics, file.path(tables_dir, "study_characteristics_quantitative.csv"))

0058

0059 outcome_summary <- effect %>%

0060 group_by(outcome_domain_formal) %>%

0061 summarise(

0062 n_studies = n_distinct(study_id),

0063 n_effects = n(),

0064 median_g = median(yi, na.rm = TRUE),

0065 min_g = min(yi, na.rm = TRUE),

0066 max_g = max(yi, na.rm = TRUE),

0067 .groups = "drop"

0068 ) %>%

0069 arrange(outcome_domain_formal)

0070 write_csv(outcome_summary, file.path(tables_dir, "outcome_domain_descriptive_summary.csv"))

0071

0072

0073 csv_files <- list.files(tables_dir, pattern = "\\.csv$", full.names = TRUE)

0074 wb <- createWorkbook()

0075 for (path in csv_files) {

0076 sheet <- substr(file_path_sans_ext(basename(path)), 1, 31)

0077 addWorksheet(wb, sheet)

0078 dat <- read_csv(path, show_col_types = FALSE)

0079 writeDataTable(wb, sheet, dat, tableStyle = "TableStyleMedium2")

0080 freezePane(wb, sheet, firstRow = TRUE)

0081 setColWidths(wb, sheet, cols = seq_along(dat), widths = "auto")

0082 }

0083 saveWorkbook(wb, file.path(tables_dir, "formal_results_tables.xlsx"), overwrite = TRUE)

0084

0085 manifest <- list.files(root, recursive = TRUE, full.names = TRUE) %>%

0086 tibble::tibble(full_path = .) %>%

0087 mutate(

0088 relative_path = gsub(paste0("^", gsub("\\\\", "/", normalizePath(root)), "/?"), "", gsub("\\\\", "/", normalizePath(full_path))),

0089 size_bytes = file.info(full_path)$size,

0090 modified_time = as.character(file.info(full_path)$mtime)

0091 ) %>%

0092 select(relative_path, size_bytes, modified_time) %>%

0093 arrange(relative_path)

0094 write_csv(manifest, file.path(metadata_dir, "output_manifest.csv"))

0095

0096 message("Formal figure data, summary tables, workbook, and manifest exported.")

0097

03_risk_of_bias_assessment.R

0001 # Calibrated RoB 2-inspired risk-of-bias assessment and visualization.

0002 # This version separates internal validity from quantitative extractability:

0003 # inability to compute an SMD is handled as reporting/extractability concern,

0004 # not automatically as high overall risk of bias.

0005

0006 options(repos = c(CRAN = "https://cran.rstudio.com/"))

0007 packages <- c("readr", "dplyr", "ggplot2", "tibble", "tidyr")

0008 missing <- packages[!(packages %in% rownames(installed.packages()))]

0009 if (length(missing) > 0) stop("Missing required R packages: ", paste(missing, collapse = ", "))

0010

0011 library(readr)

0012 library(dplyr)

0013 library(ggplot2)

0014 library(tibble)

0015 library(tidyr)

0016

0017 script_arg <- commandArgs(trailingOnly = FALSE)

0018 script_path <- normalizePath(sub("^--file=", "", script_arg[grepl("^--file=", script_arg)][1]), winslash = "/", mustWork = TRUE)

0019 root <- normalizePath(file.path(dirname(script_path), ".."), winslash = "/", mustWork = TRUE)

0020 rob_dir <- file.path(root, "risk_of_bias")

0021 dir.create(rob_dir, showWarnings = FALSE, recursive = TRUE)

0022

0023 rob <- tribble(

0024 ~study_id, ~author_year, ~synthesis, ~design, ~D1_randomization, ~D2_deviations, ~D3_missing_data, ~D4_measurement, ~D5_selection_reporting, ~D6_crossover_carryover, ~overall, ~notes,

0025 "Woolstenhulme_2004", "Woolstenhulme 2004", "quantitative", "crossover", "Some concerns", "Low", "Low", "Low", "Some concerns", "Some concerns", "Some concerns", "Older crossover report with objective outcomes; limited reporting of sequence/carryover safeguards.",

0026 "Ekstrand_2013", "Ekstrand 2013", "quantitative", "crossover", "Some concerns", "Low", "Low", "Low", "Some concerns", "Some concerns", "Some concerns", "Objective outcomes; allocation and reporting safeguards incompletely described.",

0027 "Cook_Crewther_2014", "Cook et al. 2014", "quantitative", "crossover", "Low", "Low", "Low", "Low", "Some concerns", "Low", "Low", "Counterbalanced sport-performance trial with objective outcomes; no indication of missing data or measurement bias.",

0028 "Russell_2016", "Russell 2016", "quantitative", "crossover", "Some concerns", "Low", "Some concerns", "Low", "Some concerns", "Some concerns", "Some concerns", "Trial internally relevant; some uncertainty arises from incomplete sequence/order reporting and back-calculated outcome data used for synthesis.",

0029 "Mason_2017", "Mason 2017", "quantitative", "crossover", "Low", "Low", "Low", "Low", "Some concerns", "Low", "Low", "Objective upper-body/jump outcomes and controlled crossover design; selective-reporting safeguards not fully described.",

0030 "Tsoukos_2018", "Tsoukos 2018", "quantitative", "crossover", "Some concerns", "Low", "Some concerns", "Low", "Some concerns", "Some concerns", "Some concerns", "Objective delayed outcomes; sequence/order safeguards and synthesis values were not fully direct.",

0031 "Dahl_2021", "Dahl 2021", "quantitative", "crossover", "Some concerns", "Low", "Low", "Low", "Some concerns", "Some concerns", "Some concerns", "Objective running/jump outcomes; sequence/order and selective-reporting safeguards were incompletely reported.",

0032 "Donghi_2021", "Donghi 2021", "quantitative", "crossover", "Low", "Low", "Low", "Low", "Some concerns", "Low", "Low", "Young elite soccer crossover trial with objective outcomes and complete extractable data.",

0033 "Gonzalez_Garcia_2021", "González-García 2021", "quantitative", "crossover", "Some concerns", "Low", "Low", "Low", "Some concerns", "Some concerns", "Some concerns", "Objective outcomes with extractable data; sequence/order and selective-reporting safeguards not fully described.",

0034 "Nishioka_Okada_2022", "Nishioka & Okada 2022", "quantitative", "crossover", "Some concerns", "Low", "Low", "Low", "Some concerns", "Some concerns", "Some concerns", "Objective CMJ/force-velocity outcomes and clear extractable data; sequence/order reporting was not sufficiently detailed for low D1/D6.",

0035 "Nutt_2022", "Nutt 2022", "quantitative", "crossover", "Low", "Low", "Low", "Low", "Some concerns", "Low", "Low", "Professional cricket trial with objective/cognitive outcomes; no clear missing-data or measurement concern.",

0036 "Zaras_2022", "Zaras 2022", "quantitative", "crossover", "Some concerns", "Low", "Low", "Low", "Some concerns", "Some concerns", "Some concerns", "Objective swimming/jump outcomes; sequence/carryover and selective-reporting details incompletely audited.",

0037 "Gonzalez_Garcia_2023", "González-García 2023", "quantitative", "crossover", "Some concerns", "Low", "Some concerns", "Low", "Some concerns", "Some concerns", "Some concerns", "Objective outcome; SD was derived from SWC and sequence/order reporting was incompletely detailed.",

0038 "Panteli_2024", "Panteli 2024", "quantitative", "crossover", "Some concerns", "Low", "Low", "Low", "Some concerns", "Some concerns", "Some concerns", "Objective soccer outcomes and direct extraction; sequence/order and preregistration/selective-reporting safeguards not fully described.",

0039 "Wang_2024", "Wang 2024", "quantitative", "crossover", "Some concerns", "Low", "Some concerns", "Low", "Some concerns", "Some concerns", "Some concerns", "Objective outcomes; synthesis used back-calculated values and sequence/order reporting was incompletely detailed.",

0040 "Pino_Mulero_2025", "Pino-Mulero 2025", "quantitative", "crossover", "Low", "Low", "Some concerns", "Low", "High", "Low", "Some concerns", "Internal design appears relevant, but only reported effect sizes were usable, creating high reporting/extractability concern.",

0041 "Brisola_2026", "Brisola 2026", "quantitative", "crossover", "Low", "Low", "Low", "Low", "Some concerns", "Low", "Low", "Randomized-order crossover in extracted record; objective water-polo outcomes and extractable data.",

0042 "Kolinger_2026", "Kolinger 2026", "quantitative", "crossover", "Low", "Low", "Low", "Low", "Some concerns", "Low", "Low", "Objective performance outcomes; active control was appropriate for the design and data were directly extractable.",

0043 "Saez_2007", "Saez de Villarreal 2007", "qualitative_only", "crossover/unclear", "Some concerns", "Low", "Some concerns", "Low", "Some concerns", "Some concerns", "Some concerns", "Qualitative-only because reporting prevented SMD conversion; not treated as high internal bias by default.",

0044 "McGowan_2017", "McGowan 2017", "qualitative_only", "crossover", "Low", "Low", "Some concerns", "Low", "Some concerns", "Low", "Some concerns", "Percent-change reporting limited quantitative synthesis but does not alone imply high internal bias.",

0045 "Harrison_2021", "Harrison 2021", "qualitative_only", "crossover", "Low", "Low", "Some concerns", "Low", "Some concerns", "Low", "Some concerns", "Reporting format limited SMD conversion; objective/perceptual outcomes otherwise from a controlled crossover design.",

0046 "Rud_2021", "Rud 2021", "qualitative_only", "crossover", "Low", "Low", "Some concerns", "Low", "Some concerns", "Low", "Some concerns", "Percent difference +/- 95% CI prevented SMD conversion but is not automatically high internal bias.",

0047 "Harrison_2023", "Harrison 2023", "qualitative_only", "crossover", "Low", "Low", "Some concerns", "Low", "Some concerns", "Low", "Some concerns", "Percent-of-control and Cliff's delta reporting limited synthesis compatibility.",

0048 "Harrison_2024", "Harrison 2024", "qualitative_only", "crossover", "Low", "Low", "Some concerns", "Low", "Some concerns", "Low", "Some concerns", "Percent-of-control and Cliff's delta reporting limited synthesis compatibility.",

0049 "Gonzalez_Garcia_caffeine_2023", "González-García caffeine 2023", "qualitative_only", "crossover", "Low", "Low", "Some concerns", "Low", "Some concerns", "Low", "Some concerns", "Priming-alone data were not convertible to raw condition SMD; internal bias not judged high on that basis alone.",

0050 "Holmberg_2026", "Holmberg 2026", "qualitative_only", "crossover", "Low", "Low", "Some concerns", "Low", "Some concerns", "Low", "Some concerns", "Model-derived marginal effects lacked raw SD for SMD conversion; objective outcomes and controlled design support some-concerns rather than high-risk rating."

0051 )

0052

0053 domain_cols <- c(

0054 "D1_randomization", "D2_deviations", "D3_missing_data",

0055 "D4_measurement", "D5_selection_reporting",

0056 "D6_crossover_carryover", "overall"

0057 )

0058

0059 write_csv(rob, file.path(rob_dir, "risk_of_bias_domain_judgements.csv"))

0060

0061 summary <- rob %>%

0062 pivot_longer(all_of(domain_cols), names_to = "domain", values_to = "judgement") %>%

0063 count(synthesis, domain, judgement) %>%

0064 group_by(synthesis, domain) %>%

0065 mutate(percent = 100 * n / sum(n)) %>%

0066 ungroup()

0067 write_csv(summary, file.path(rob_dir, "risk_of_bias_summary.csv"))

0068

0069 if ("openxlsx" %in% rownames(installed.packages())) {

0070 library(openxlsx)

0071 wb <- createWorkbook()

0072 addWorksheet(wb, "domain_judgements")

0073 writeDataTable(wb, "domain_judgements", rob, tableStyle = "TableStyleMedium2")

0074 freezePane(wb, "domain_judgements", firstRow = TRUE)

0075 setColWidths(wb, "domain_judgements", cols = 1:ncol(rob), widths = "auto")

0076 addWorksheet(wb, "summary")

0077 writeDataTable(wb, "summary", summary, tableStyle = "TableStyleMedium2")

0078 freezePane(wb, "summary", firstRow = TRUE)

0079 setColWidths(wb, "summary", cols = 1:ncol(summary), widths = "auto")

0080 saveWorkbook(wb, file.path(rob_dir, "risk_of_bias_assessment.xlsx"), overwrite = TRUE)

0081 }

0082

0083 level_cols <- c("Low" = "#2ca25f", "Some concerns" = "#fdae61", "High" = "#de2d26")

0084 domain_labs <- c(

0085 D1_randomization = "D1 Randomization",

0086 D2_deviations = "D2 Deviations",

0087 D3_missing_data = "D3 Missing outcome data",

0088 D4_measurement = "D4 Measurement",

0089 D5_selection_reporting = "D5 Selection/reporting",

0090 D6_crossover_carryover = "D6 Carryover",

0091 overall = "Overall"

0092 )

0093

0094 plot_summary <- summary %>%

0095 mutate(

0096 domain = factor(domain, levels = names(domain_labs), labels = domain_labs),

0097 judgement = factor(judgement, levels = c("Low", "Some concerns", "High")),

0098 synthesis_label = recode(

0099 synthesis,

0100 quantitative = "Quantitative meta-analysis",

0101 qualitative_only = "SMD-incompatible qualitative-only eligible studies"

0102 )

0103 )

0104

0105 p <- ggplot(plot_summary, aes(x = domain, y = percent, fill = judgement)) +

0106 geom_col(width = 0.75, color = "white") +

0107 coord_flip() +

0108 facet_wrap(~ synthesis_label, ncol = 1) +

0109 scale_fill_manual(values = level_cols, drop = FALSE) +

0110 labs(x = NULL, y = "Studies (%)", fill = "Judgement",

0111 title = "Risk-of-bias summary",

0112 subtitle = "RoB 2-inspired judgments calibrated for crossover sport-performance trials") +

0113 theme_minimal(base_size = 12) +

0114 theme(panel.grid.major.y = element_blank())

0115 ggsave(file.path(rob_dir, "risk_of_bias_summary.png"), p, width = 9, height = 7, dpi = 300)

0116

0117 traffic <- rob %>%

0118 select(author_year, synthesis, all_of(domain_cols)) %>%

0119 pivot_longer(all_of(domain_cols), names_to = "domain", values_to = "judgement") %>%

0120 mutate(

0121 domain = factor(domain, levels = names(domain_labs), labels = domain_labs),

0122 judgement = factor(judgement, levels = c("Low", "Some concerns", "High")),

0123 author_year = factor(author_year, levels = rev(rob$author_year))

0124 )

0125

0126 p2 <- ggplot(traffic, aes(x = domain, y = author_year, fill = judgement)) +

0127 geom_tile(color = "white", linewidth = 0.4) +

0128 scale_fill_manual(values = level_cols, drop = FALSE) +

0129 labs(x = NULL, y = NULL, fill = "Judgement",

0130 title = "Risk-of-bias traffic-light plot",

0131 subtitle = "Extractability concerns are not treated as high internal bias by default") +

0132 theme_minimal(base_size = 10) +

0133 theme(axis.text.x = element_text(angle = 35, hjust = 1),

0134 panel.grid = element_blank())

0135 ggsave(file.path(rob_dir, "risk_of_bias_traffic_light.png"), p2, width = 11, height = 9, dpi = 300)

0136

0137 readme <- c(

0138 "# Risk of Bias Assessment",

0139 "",

0140 "This folder contains a calibrated RoB 2-inspired assessment for the formal delayed priming review.",

0141 "",

0142 "The revised assessment follows a top-journal sport meta-analysis logic: internal validity, quantitative extractability, comparator compatibility, and reporting completeness are related but not identical. Studies were not rated high risk solely because their published data could not be converted to SMD.",

0143 "",

0144 "Domains:",

0145 "",

0146 "- D1: randomization, allocation, or period-order information.",

0147 "- D2: deviations from intended interventions.",

0148 "- D3: missing outcome data.",

0149 "- D4: outcome measurement.",

0150 "- D5: selection of the reported result and reporting completeness.",

0151 "- D6: crossover carryover, washout, and period effects.",

0152 "",

0153 "Interpretation:",

0154 "",

0155 "- Objective performance measurement was usually low risk.",

0156 "- Most concerns reflect incomplete reporting, absence of preregistered primary outcomes, or incomplete variance information.",

0157 "- High risk was reserved for clear design-level or reporting-level threats that could materially bias the synthesized effect.",

0158 "",

0159 "Files:",

0160 "",

0161 "- `risk_of_bias_domain_judgements.csv`",

0162 "- `risk_of_bias_summary.csv`",

0163 "- `risk_of_bias_assessment.xlsx`",

0164 "- `risk_of_bias_summary.png`",

0165 "- `risk_of_bias_traffic_light.png`"

0166 )

0167 writeLines(readme, file.path(rob_dir, "README.md"), useBytes = TRUE)

0168

0169 message("Calibrated risk-of-bias assessment exported.")

0170

04_grade_assessment.R

0001 # GRADE certainty-of-evidence assessment for the formal manuscript.

0002

0003 options(repos = c(CRAN = "https://cran.rstudio.com/"))

0004 packages <- c("readr", "dplyr", "openxlsx", "tibble")

0005 missing <- packages[!(packages %in% rownames(installed.packages()))]

0006 if (length(missing) > 0) install.packages(missing, dependencies = TRUE)

0007

0008 library(readr)

0009 library(dplyr)

0010 library(openxlsx)

0011 library(tibble)

0012

0013 script_arg <- commandArgs(trailingOnly = FALSE)

0014 script_path <- normalizePath(sub("^--file=", "", script_arg[grepl("^--file=", script_arg)][1]), winslash = "/", mustWork = TRUE)

0015 root <- normalizePath(file.path(dirname(script_path), ".."), winslash = "/", mustWork = TRUE)

0016 grade_dir <- file.path(root, "grade")

0017 dir.create(grade_dir, showWarnings = FALSE, recursive = TRUE)

0018

0019 sof <- tribble(

0020 ~outcome, ~studies, ~effect_sizes, ~effect, ~risk_of_bias, ~inconsistency, ~indirectness, ~imprecision, ~publication_bias, ~certainty, ~interpretation,

0021 "Overall athletic performance", 18, 46, "g = 0.232 (95% CI 0.092 to 0.372); PI -0.265 to 0.730", "Some concerns, not primary downgrade", "Serious", "Not serious", "Not serious", "Undetected but cannot be excluded", "Low", "Small average benefit; certainty mainly limited by heterogeneous effects and a prediction interval crossing no effect.",

0022 "Strength/power outcomes", 17, 26, "g = 0.202 (95% CI 0.083 to 0.321)", "Some concerns", "Not serious", "Not serious", "Serious", "Undetected but cannot be excluded", "Low", "Most consistent domain-level average estimate, but certainty is limited because subgroup contrasts were exploratory and did not establish differential effectiveness.",

0023 "Speed/agility outcomes", 7, 9, "g = 0.337 (95% CI -0.143 to 0.818)", "Some concerns", "Serious", "Not serious", "Serious", "Undetected but cannot be excluded", "Very low", "Potentially larger effect but highly uncertain because CI crosses no effect and heterogeneity is high.",

0024 "Sport-specific outcomes", 8, 11, "g = 0.075 (95% CI -0.117 to 0.268)", "Some concerns", "Not serious", "Serious", "Serious", "Undetected but cannot be excluded", "Very low", "Evidence is compatible with little or no average sport-specific benefit.",

0025 "Intervals of at least 6 h", 12, 32, "g = 0.288 (95% CI 0.086 to 0.490); PI -0.348 to 0.924", "Some concerns, not primary downgrade", "Serious", "Not serious", "Not serious", "Undetected but cannot be excluded", "Low", "Stronger delayed signal, but prediction interval remains wide and crosses no effect."

0026 )

0027

0028 write_csv(sof, file.path(grade_dir, "grade_summary_of_findings.csv"))

0029

0030 wb <- createWorkbook()

0031 addWorksheet(wb, "summary_of_findings")

0032 writeDataTable(wb, "summary_of_findings", sof, tableStyle = "TableStyleMedium2")

0033 freezePane(wb, "summary_of_findings", firstRow = TRUE)

0034 setColWidths(wb, "summary_of_findings", cols = 1:ncol(sof), widths = "auto")

0035 saveWorkbook(wb, file.path(grade_dir, "grade_summary_of_findings.xlsx"), overwrite = TRUE)

0036

0037 readme <- c(

0038 "# GRADE Certainty Assessment",

0039 "",

0040 "This folder contains the formal GRADE-style certainty assessment used in the manuscript.",

0041 "",

0042 "The assessment starts randomized/crossover trials at high certainty, then downgrades for risk of bias, inconsistency, indirectness, imprecision, and publication bias.",

0043 "",

0044 "Risk of bias was calibrated to separate internal validity from quantitative extractability. Most concerns reflect incomplete reporting rather than clear evidence of biased objective performance measurement.",

0045 "",

0046 "Primary conclusion:",

0047 "",

0048 "- Overall athletic performance: Low certainty.",

0049 "- Strength/power outcomes: Low certainty. Although this was the most consistent domain-level average estimate, subgroup contrasts were exploratory and do not establish differential effectiveness.",

0050 "- Speed/agility and sport-specific subgroup evidence: Very low certainty.",

0051 "",

0052 "Files:",

0053 "",

0054 "- `grade_summary_of_findings.csv`",

0055 "- `grade_summary_of_findings.xlsx`"

0056 )

0057 writeLines(readme, file.path(grade_dir, "README.md"), useBytes = TRUE)

0058

0059 message("GRADE assessment exported.")

0060

05_crossover_correlation_sensitivity.R

0001 # Sensitivity analysis for assumed within-participant correlation in repeated-measures SMDs.

0002

0003 options(repos = c(CRAN = "https://cran.rstudio.com/"))

0004 packages <- c("metafor", "readr", "dplyr", "tibble")

0005 missing <- packages[!(packages %in% rownames(installed.packages()))]

0006 if (length(missing) > 0) stop("Missing required R packages: ", paste(missing, collapse = ", "))

0007

0008 library(metafor)

0009 library(readr)

0010 library(dplyr)

0011 library(tibble)

0012 has_clubSandwich <- "clubSandwich" %in% rownames(installed.packages())

0013 if (has_clubSandwich) library(clubSandwich)

0014

0015 script_arg <- commandArgs(trailingOnly = FALSE)

0016 script_path <- normalizePath(sub("^--file=", "", script_arg[grepl("^--file=", script_arg)][1]), winslash = "/", mustWork = TRUE)

0017 root <- normalizePath(file.path(dirname(script_path), "..", ".."), winslash = "/", mustWork = TRUE)

0018 data_path <- file.path(root, "data", "extracted_data_master.csv")

0019 out_dir <- file.path(root, "formal_outputs")

0020 tab_dir <- file.path(out_dir, "tables")

0021 data_dir <- file.path(out_dir, "figure_data")

0022 dir.create(tab_dir, showWarnings = FALSE, recursive = TRUE)

0023 dir.create(data_dir, showWarnings = FALSE, recursive = TRUE)

0024

0025 raw <- read_csv(data_path, show_col_types = FALSE) %>%

0026 filter(include_main == 1, direction %in% c("higher_better", "lower_better")) %>%

0027 mutate(

0028 study_id = as.character(study_id),

0029 effect_id = as.character(effect_id),

0030 design = as.character(design),

0031 extraction_status = as.character(extraction_status)

0032 )

0033

0034 calc_cross_es <- function(df, r = 0.5) {

0035 df %>%

0036 mutate(

0037 sd_within = sqrt(sd_priming^2 + sd_control^2 - 2 * r * sd_priming * sd_control),

0038 d_rm = (mean_priming - mean_control) / sd_within,

0039 J = 1 - (3 / (4 * n_priming - 5)),

0040 yi_calc = J * d_rm,

0041 vi_calc = (1 / n_priming + yi_calc^2 / (2 * n_priming)) * J^2,

0042 es_method = paste0("crossover rm-SMD; assumed r=", r)

0043 )

0044 }

0045

0046 calc_prepost_es <- function(df, r = 0.5) {

0047 df %>%

0048 mutate(

0049 d_change = (mean_priming - mean_control) / sd_control,

0050 J = 1 - (3 / (4 * n_priming - 5)),

0051 yi_calc = J * d_change,

0052 vi_calc = (2 * (1 - r) / n_priming + yi_calc^2 / (2 * n_priming)) * J^2,

0053 es_method = paste0("pre-post SMD; assumed r=", r)

0054 )

0055 }

0056

0057 calc_ind_es <- function(df) {

0058 esc <- escalc(

0059 measure = "SMD",

0060 m1i = mean_priming, sd1i = sd_priming, n1i = n_priming,

0061 m2i = mean_control, sd2i = sd_control, n2i = n_control,

0062 data = df

0063 )

0064 df$yi_calc <- esc$yi

0065 df$vi_calc <- esc$vi

0066 df$es_method <- "independent-groups SMD"

0067 df

0068 }

0069

0070 make_es <- function(r_assumed) {

0071 dat_es <- raw %>% mutate(yi = NA_real_, vi = NA_real_, es_method = NA_character_)

0072

0073 reported_idx <- which(!is.na(dat_es$yi_reported) & !is.na(dat_es$vi_reported))

0074 if (length(reported_idx) > 0) {

0075 dat_es$yi[reported_idx] <- dat_es$yi_reported[reported_idx]

0076 dat_es$vi[reported_idx] <- dat_es$vi_reported[reported_idx]

0077 dat_es$es_method[reported_idx] <- "reported ES; approximate variance"

0078 }

0079

0080 remaining <- which(is.na(dat_es$yi))

0081 pp_idx <- remaining[grepl("pre-post|repeated measures", dat_es$design[remaining], ignore.case = TRUE)]

0082 if (length(pp_idx) > 0) {

0083 tmp <- calc_prepost_es(dat_es[pp_idx, ], r = r_assumed)

0084 dat_es[pp_idx, c("yi", "vi", "es_method")] <- tmp[, c("yi_calc", "vi_calc", "es_method")]

0085 }

0086

0087 remaining <- which(is.na(dat_es$yi))

0088 cross_idx <- remaining[dat_es$design[remaining] == "crossover"]

0089 if (length(cross_idx) > 0) {

0090 tmp <- calc_cross_es(dat_es[cross_idx, ], r = r_assumed)

0091 dat_es[cross_idx, c("yi", "vi", "es_method")] <- tmp[, c("yi_calc", "vi_calc", "es_method")]

0092 }

0093

0094 remaining <- which(is.na(dat_es$yi))

0095 if (length(remaining) > 0) {

0096 tmp <- calc_ind_es(dat_es[remaining, ])

0097 dat_es[remaining, c("yi", "vi", "es_method")] <- tmp[, c("yi_calc", "vi_calc", "es_method")]

0098 }

0099

0100 dat_es %>%

0101 mutate(

0102 yi = if_else(direction == "lower_better" & is.na(yi_reported), -yi, yi),

0103 vi = as.numeric(vi)

0104 ) %>%

0105 filter(!is.na(yi), !is.na(vi), vi > 0)

0106 }

0107

0108 fit_one <- function(r_assumed) {

0109 df <- make_es(r_assumed)

0110 res <- rma.mv(yi = yi, V = vi, random = ~ 1 | study_id / effect_id,

0111 method = "REML", test = "t", data = df)

0112 pred <- predict(res)

0113 robust <- if (has_clubSandwich) {

0114 tryCatch(

0115 coef_test(res, vcov = "CR2", cluster = df$study_id, test = "Satterthwaite"),

0116 error = function(e) tibble(SE = NA_real_, p_Satt = NA_real_)

0117 )

0118 } else {

0119 tibble(SE = NA_real_, p_Satt = NA_real_)

0120 }

0121 tibble(

0122 assumed_r = r_assumed,

0123 k_studies = n_distinct(df$study_id),

0124 m_effects = nrow(df),

0125 g = as.numeric(coef(res)),

0126 ci_lb = res$ci.lb,

0127 ci_ub = res$ci.ub,

0128 p = res$pval,

0129 pi_lb = pred$cr.lb,

0130 pi_ub = pred$cr.ub,

0131 tau2_level3 = res$sigma2[1],

0132 tau2_level2 = res$sigma2[2],

0133 robust_se = robust$SE[1],

0134 robust_p = robust$p_Satt[1]

0135 )

0136 }

0137

0138 out <- bind_rows(lapply(c(0.30, 0.50, 0.70, 0.90), fit_one))

0139 write_csv(out, file.path(tab_dir, "crossover_correlation_sensitivity.csv"))

0140 write_csv(out, file.path(data_dir, "crossover_correlation_sensitivity.csv"))

0141

0142 message("Crossover correlation sensitivity exported.")

0143

generate_fig2_from_local_data.R; generate_fig3_from_local_data.R

0001 from pathlib import Path

0002 import csv

0003 import shutil

0004

0005 import pandas as pd

0006 from PIL import Image, ImageDraw, ImageFont

0007

0008

0009 ROOT = Path(__file__).resolve().parents[2]

0010 OUT = ROOT / "formal_outputs"

0011 FIG = OUT / "figures"

0012 FIG_DATA = OUT / "figure_data"

0013 TABLES = OUT / "tables"

0014 ROB = OUT / "risk_of_bias"

0015 SUPP_FIG = ROOT / "supplementary_materials" / "01_figures"

0016 SUPP_DATA = ROOT / "supplementary_materials" / "04_data"

0017

0018 for path in [FIG, FIG_DATA, TABLES, ROB, SUPP_FIG, SUPP_DATA]:

0019 path.mkdir(parents=True, exist_ok=True)

0020

0021

0022 def font(size=28, bold=False):

0023 candidates = [

0024 r"C:\Windows\Fonts\arialbd.ttf" if bold else r"C:\Windows\Fonts\arial.ttf",

0025 r"C:\Windows\Fonts\timesbd.ttf" if bold else r"C:\Windows\Fonts\times.ttf",

0026 ]

0027 for candidate in candidates:

0028 if Path(candidate).exists():

0029 return ImageFont.truetype(candidate, size=size)

0030 return ImageFont.load_default()

0031

0032

0033 def draw_wrapped(draw, xy, text, fnt, fill, max_width, line_gap=6):

0034 words = str(text).split()

0035 lines, line = [], ""

0036 for word in words:

0037 test = word if not line else f"{line} {word}"

0038 if draw.textbbox((0, 0), test, font=fnt)[2] <= max_width:

0039 line = test

0040 else:

0041 if line:

0042 lines.append(line)

0043 line = word

0044 if line:

0045 lines.append(line)

0046 x, y = xy

0047 for line in lines:

0048 draw.text((x, y), line, font=fnt, fill=fill)

0049 y += fnt.size + line_gap

0050 return y

0051

0052

0053 def regenerate_prisma():

0054 counts = [

0055 ("Records identified", 3095, "EndNote raw export before deduplication"),

0056 ("Duplicate records removed", 1107, "Deduplication by title/DOI/PMID"),

0057 ("Records screened after deduplication", 1988, "Title/abstract screening"),

0058 ("Records excluded at title/abstract", 1776, "Not delayed exercise-based priming / not eligible"),

0059 ("Reports assessed for eligibility", 212, "Full-text/web-assisted eligibility assessment"),

0060 ("Reports excluded at eligibility", 186, "Did not meet PICOS, publication type, comparator, intervention, or data criteria"),

0061 ("Studies included in qualitative synthesis", 26, "18 quantitative + 8 SMD-incompatible narrative-only"),

0062 ("Studies included in quantitative synthesis", 18, "46 effect sizes"),

0063 ]

0064 with (FIG_DATA / "prisma_formal_counts.csv").open("w", newline="", encoding="utf-8") as handle:

0065 writer = csv.writer(handle)

0066 writer.writerow(["Step", "Count", "Notes"])

0067 writer.writerows(counts)

0068

0069 width, height = 1800, 2350

0070 img = Image.new("RGB", (width, height), "white")

0071 draw = ImageDraw.Draw(img)

0072 title_f = font(42, True)

0073 box_f = font(30)

0074 small_f = font(25)

0075 dark, line, fill, edge = "#1f2937", "#4b5563", "#f8fafc", "#111827"

0076

0077 draw.text((width // 2, 70), "PRISMA 2020 Flow Diagram", anchor="mm", font=title_f, fill=dark)

0078 boxes = [

0079 (250, 170, 1150, 320, "Records identified\n(n = 3,095)"),

0080 (250, 420, 1150, 560, "Duplicate records removed\n(n = 1,107)"),

0081 (250, 700, 1150, 850, "Records screened after deduplication\n(n = 1,988)"),

0082 (1220, 900, 1760, 1040, "Records excluded at title/abstract\n(n = 1,776)"),

0083 (250, 1120, 1150, 1270, "Reports assessed for eligibility\n(n = 212)"),

0084 (1220, 1340, 1760, 1530, "Reports excluded at eligibility\n(n = 186)\nPICOS, publication type, comparator,\nintervention, or data criteria"),

0085 (250, 1650, 1150, 1790, "Studies included in qualitative synthesis\n(n = 26)"),

0086 (250, 1880, 1150, 2020, "Studies included in quantitative synthesis\n(n = 18; 46 effect sizes)"),

0087 (250, 2090, 1150, 2260, "Narrative-only studies\n(n = 8; SMD-incompatible)"),

0088 ]

0089 for x1, y1, x2, y2, text in boxes:

0090 draw.rounded_rectangle((x1, y1, x2, y2), radius=18, fill=fill, outline=edge, width=3)

0091 lines = text.split("\n")

0092 total = len(lines) * 34

0093 y = (y1 + y2) / 2 - total / 2

0094 for line_text in lines:

0095 draw.text(((x1 + x2) / 2, y), line_text, anchor="ma", font=box_f if len(line_text) < 58 else small_f, fill=dark)

0096 y += 34

0097

0098 def arrow(x1, y1, x2, y2):

0099 draw.line((x1, y1, x2, y2), fill=line, width=5)

0100 if abs(x2 - x1) > abs(y2 - y1):

0101 if x2 > x1:

0102 draw.polygon([(x2, y2), (x2 - 28, y2 - 15), (x2 - 28, y2 + 15)], fill=line)

0103 else:

0104 draw.polygon([(x2, y2), (x2 + 28, y2 - 15), (x2 + 28, y2 + 15)], fill=line)

0105 elif y2 > y1:

0106 draw.polygon([(x2, y2), (x2 - 15, y2 - 28), (x2 + 15, y2 - 28)], fill=line)

0107 else:

0108 draw.polygon([(x2, y2), (x2 - 28, y2 - 15), (x2 - 28, y2 + 15)], fill=line)

0109

0110 def elbow_arrow(x1, y1, x2, y2):

0111 mid_x = min(max(x1 + 80, (x1 + x2) / 2), x2 - 20)

0112 draw.line((x1, y1, mid_x, y1, mid_x, y2, x2, y2), fill=line, width=5)

0113 draw.polygon([(x2, y2), (x2 - 28, y2 - 15), (x2 - 28, y2 + 15)], fill=line)

0114

0115 arrow(700, 320, 700, 420)

0116 arrow(700, 560, 700, 700)

0117 arrow(700, 850, 700, 1120)

0118 elbow_arrow(1150, 775, 1220, 970)

0119 arrow(700, 1270, 700, 1650)

0120 elbow_arrow(1150, 1195, 1220, 1435)

0121 arrow(700, 1790, 700, 1880)

0122 arrow(700, 2020, 700, 2090)

0123

0124 out = FIG / "prisma_2020_flow_diagram.png"

0125 img.save(out, dpi=(300, 300))

0126 shutil.copy2(out, SUPP_FIG / "SF1_prisma_2020_flow_diagram.png")

0127

0128

0129 def regenerate_forest():

0130 data = pd.read_csv(TABLES / "study_level_aggregated_effects.csv")

0131 data = data.sort_values(["year", "author"])

0132 model = pd.read_csv(TABLES / "overall_and_sensitivity_models.csv")

0133 main = model.loc[model["analysis"] == "Main formal model"].iloc[0]

0134 width, height = 1400, 1300

0135 left, right = 430, 1120

0136 top, row_h = 115, 52

0137 axis_y = height - 150

0138 x_min, x_max = -0.8, 1.4

0139

0140 img = Image.new("RGB", (width, height), "white")

0141 draw = ImageDraw.Draw(img)

0142 title_f, label_f, small_f = font(34, True), font(22), font(20)

0143 draw.text((width // 2, 35), "Study-level forest plot", anchor="ma", font=title_f, fill="#111827")

0144 draw.text((width // 2, 77), "Hedges' g; positive values favor delayed priming", anchor="ma", font=label_f, fill="#374151")

0145

0146 def xmap(v):

0147 return left + (float(v) - x_min) / (x_max - x_min) * (right - left)

0148

0149 for tick in [-0.5, 0, 0.5, 1.0]:

0150 x = xmap(tick)

0151 draw.line((x, top - 15, x, axis_y), fill="#e5e7eb", width=2)

0152 draw.text((x, axis_y + 18), f"{tick:g}", anchor="ma", font=small_f, fill="#4b5563")

0153 draw.line((xmap(0), top - 15, xmap(0), axis_y), fill="#6b7280", width=3)

0154

0155 draw.text((40, top - 40), "Study", font=label_f, fill="#111827")

0156 draw.text((right + 35, top - 40), "g [95% CI]", font=label_f, fill="#111827")

0157

0158 for idx, row in enumerate(data.itertuples(index=False)):

0159 y = top + idx * row_h

0160 g = float(row.yi)

0161 se = float(row.vi) ** 0.5

0162 lo, hi = g - 1.96 * se, g + 1.96 * se

0163 draw.text((40, y - 10), f"{row.author} ({int(row.year)})", font=small_f, fill="#1f2937")

0164 draw.line((xmap(max(lo, x_min)), y, xmap(min(hi, x_max)), y), fill="#2563eb", width=4)

0165 draw.ellipse((xmap(g) - 7, y - 7, xmap(g) + 7, y + 7), fill="#1d4ed8")

0166 draw.text((right + 35, y - 12), f"{g:.2f} [{lo:.2f}, {hi:.2f}]", font=small_f, fill="#1f2937")

0167

0168 pooled_y = axis_y - 50

0169 g, lo, hi = float(main.g), float(main.ci_lb), float(main.ci_ub)

0170 diamond = [(xmap(g), pooled_y - 16), (xmap(hi), pooled_y), (xmap(g), pooled_y + 16), (xmap(lo), pooled_y)]

0171 draw.polygon(diamond, fill="#dc2626")

0172 draw.text((40, pooled_y - 13), "Three-level pooled estimate", font=label_f, fill="#111827")

0173 draw.text((right + 35, pooled_y - 13), f"{g:.2f} [{lo:.2f}, {hi:.2f}]", font=label_f, fill="#111827")

0174

0175 draw.line((left, axis_y, right, axis_y), fill="#111827", width=3)

0176 draw.text(((left + right) / 2, height - 75), "Hedges' g", anchor="ma", font=label_f, fill="#111827")

0177 out = FIG / "forest_study_level.png"

0178 img.save(out, dpi=(300, 300))

0179

0180

0181 def regenerate_rob_summary():

0182 rows = [

0183 ("qualitative_only", "Overall", 0, 8, 0),

0184 ("qualitative_only", "D6 Carryover", 7, 1, 0),

0185 ("qualitative_only", "D5 Selection/reporting", 0, 8, 0),

0186 ("qualitative_only", "D4 Measurement", 8, 0, 0),

0187 ("qualitative_only", "D3 Missing outcome data", 0, 8, 0),

0188 ("qualitative_only", "D2 Deviations", 8, 0, 0),

0189 ("qualitative_only", "D1 Randomization", 7, 1, 0),

0190 ("quantitative", "Overall", 6, 12, 0),

0191 ("quantitative", "D6 Carryover", 7, 11, 0),

0192 ("quantitative", "D5 Selection/reporting", 0, 17, 1),

0193 ("quantitative", "D4 Measurement", 18, 0, 0),

0194 ("quantitative", "D3 Missing outcome data", 13, 5, 0),

0195 ("quantitative", "D2 Deviations", 18, 0, 0),

0196 ("quantitative", "D1 Randomization", 7, 11, 0),

0197 ]

0198 with (FIG_DATA / "main_rob_summary_data.csv").open("w", newline="", encoding="utf-8") as handle:

0199 writer = csv.writer(handle)

0200 writer.writerow(["synthesis", "domain", "Low", "Some concerns", "High"])

0201 writer.writerows(rows)

0202

0203 width, height = 2700, 2450

0204 img = Image.new("RGB", (width, height), "white")

0205 draw = ImageDraw.Draw(img)

0206 title_f, sub_f, label_f, small_f = font(58), font(42), font(31), font(28)

0207 colors = {"Low": "#2ca25f", "Some concerns": "#fdae61", "High": "#de2d26"}

0208 draw.text((485, 40), "Risk-of-bias summary", font=title_f, fill="#111111")

0209 draw.text((485, 130), "RoB 2 principles calibrated for crossover sport-performance trials", font=sub_f, fill="#111111")

0210

0211 left, right = 560, 2135

0212 bar_h = 48

0213 start_y = 285

0214 gap = 66

0215 panel_gap = 115

0216

0217 def draw_panel(synthesis, y0):

0218 panel_titles = {

0219 "quantitative": "Quantitative meta-analysis",

0220 "qualitative_only": "SMD-incompatible qualitative-only eligible studies",

0221 }

0222 panel_rows = [r for r in rows if r[0] == synthesis]

0223 n_total = {

0224 "qualitative_only": 8,

0225 "quantitative": 18,

0226 }[synthesis]

0227 draw.text(((left + right) / 2, y0 - 60), panel_titles[synthesis], anchor="ma", font=sub_f, fill="#222222")

0228 for i, (_, domain, low, some, high) in enumerate(panel_rows):

0229 y = y0 + i * gap

0230 draw.text((455, y + bar_h / 2), domain, anchor="rm", font=small_f, fill="#555555")

0231 x = left

0232 for label, count in [("High", high), ("Some concerns", some), ("Low", low)]:

0233 if count <= 0:

0234 continue

0235 w = (right - left) * count / n_total

0236 draw.rectangle((x, y, x + w, y + bar_h), fill=colors[label], outline="white", width=4)

0237 x += w

0238 for pct in [0, 25, 50, 75, 100]:

0239 gx = left + (right - left) * pct / 100

0240 draw.line((gx, y - 12, gx, y + bar_h + 12), fill="#e5e7eb", width=2)

0241 return y0 + len(panel_rows) * gap

0242

0243 y_after = draw_panel("quantitative", start_y)

0244 y_after = draw_panel("qualitative_only", y_after + panel_gap)

0245

0246 axis_y = height - 150

0247 draw.line((left, axis_y, right, axis_y), fill="#111111", width=3)

0248 for pct in [0, 25, 50, 75, 100]:

0249 x = left + (right - left) * pct / 100

0250 draw.text((x, axis_y + 24), str(pct), anchor="ma", font=small_f, fill="#555555")

0251 draw.text(((left + right) / 2, height - 62), "Studies (%)", anchor="ma", font=sub_f, fill="#111111")

0252

0253 lx, ly = 2290, 760

0254 draw.text((lx, ly - 70), "Judgement", font=sub_f, fill="#111111")

0255 for i, label in enumerate(["Low", "Some concerns", "High"]):

0256 y = ly + i * 80

0257 draw.rectangle((lx, y, lx + 62, y + 62), fill=colors[label])

0258 draw.text((lx + 90, y + 8), label, font=label_f, fill="#111111")

0259

0260 out = ROB / "risk_of_bias_summary.png"

0261 img.save(out, dpi=(300, 300))

0262 shutil.copy2(out, SUPP_FIG / "SF8_risk_of_bias_summary.png")

0263

0264

0265 def export_main_tables():

0266 model = pd.read_csv(TABLES / "overall_and_sensitivity_models.csv")

0267 keep = ["Main formal model", "≥6 h only", "One preferred outcome per study"]

0268 table1 = model[model["analysis"].isin(keep)].copy()

0269 table1["Analysis"] = pd.Categorical(table1["analysis"], keep, ordered=True)

0270 table1 = table1.sort_values("Analysis")

0271 table1_out = pd.DataFrame({

0272 "Analysis": table1["analysis"],

0273 "k": table1["k_studies"].astype(int),

0274 "m": table1["m_effects"].astype(int),

0275 "g": table1["g"].map(lambda x: f"{x:.3f}"),

0276 "95% CI": table1.apply(lambda r: f"{r.ci_lb:.3f}, {r.ci_ub:.3f}", axis=1),

0277 "p": table1["p"].map(lambda x: "< .001" if x < 0.001 else f"{x:.3f}".replace("0.", ".")),

0278 "PI": table1.apply(lambda r: f"{r.pi_lb:.3f}, {r.pi_ub:.3f}", axis=1),

0279 "CR2 p": table1["robust_p"].map(lambda x: "< .001" if x < 0.001 else f"{x:.3f}".replace("0.", ".")),

0280 })

0281 table2_out = pd.DataFrame([

0282 ["Quantitative meta-analysis studies", 6, 12, 0],

0283 ["SMD-incompatible qualitative-only studies", 0, 8, 0],

0284 ], columns=["Synthesis", "Low", "Some concerns", "High"])

0285 table1_out.to_csv(TABLES / "main_manuscript_table1.csv", index=False, encoding="utf-8-sig")

0286 table2_out.to_csv(TABLES / "main_manuscript_table2.csv", index=False, encoding="utf-8-sig")

0287

0288

0289 if __name__ == "__main__":

0290 regenerate_prisma()

0291 regenerate_forest()

0292 regenerate_rob_summary()

0293 export_main_tables()

0294 print("Regenerated main manuscript figures and table data for 18 studies / 46 effect sizes.")

0295
