# Supplementary material for: Does delayed exercise-based priming improve subsequent athletic performance? A systematic review and multilevel meta-analysis
Source: PLoS One. 2026 Jul 30;21(7):e0354720. doi: 10.1371/journal.pone.0354720 (PMC13422850; doi:10.1371/journal.pone.0354720)
Supplement: S2 Table — (DOCX) [file pone.0354720.s005.docx]

**S2 Table. Full-text exclusion categories.**

Note. Categories are based on the final full-text exclusion log. A total of 212 full-text reports were assessed. Of these, 186 reports were excluded and 26 studies were included in the qualitative synthesis. Detailed exclusion reasons were assigned to standard PRISMA-style grouped reasons used in Fig 1.

Grouped totals: Other PICOS mismatch (n = 68), wrong population or setting (n = 65), wrong intervention or timing (n = 34), wrong outcome or topic (n = 12), wrong comparator or design (n = 3), and not original peer-reviewed research (n = 4).

| Detailed exclusion reason | Grouped PRISMA reason | Reports excluded (n) |
| --- | --- | --- |
| Other PICOS mismatch | Other PICOS mismatch | 68 |
| Non-human / in vitro study | Wrong population or setting | 61 |
| Clinical/injury/biomechanical non-athlete study | Wrong population or setting | 2 |
| Clinical/basic preconditioning topic | Wrong population or setting | 2 |
| Non-exercise priming or non-eligible modality | Wrong intervention or timing | 10 |
| Acute PAP/PAPE only (<1 h or no delayed component) | Wrong intervention or timing | 7 |
| Acute PAP/PAPE only | Wrong intervention or timing | 6 |
| Insufficient delayed priming signal at full-text triage | Wrong intervention or timing | 5 |
| Acute/non-eligible potentiation strategy | Wrong intervention or timing | 1 |
| Nutrition/recovery intervention | Wrong intervention or timing | 1 |
| Acute/same-day PAPE competition warm-up, not delayed priming | Wrong intervention or timing | 1 |
| No delayed priming component | Wrong intervention or timing | 1 |
| Recovery intervention | Wrong intervention or timing | 1 |
| Immediate loaded warm-up | Wrong intervention or timing | 1 |
| Wrong topic or non-performance outcome | Wrong outcome or topic | 7 |
| Wrong outcome | Wrong outcome or topic | 2 |
| Wrong outcome/topic | Wrong outcome or topic | 1 |
| Wrong topic | Wrong outcome or topic | 1 |
| Descriptive warm-up strategy study | Wrong outcome or topic | 1 |
| No eligible intervention comparison | Wrong comparator or design | 1 |
| No eligible comparator | Wrong comparator or design | 1 |
| Non-randomized observational match comparison | Wrong comparator or design | 1 |
| Review/commentary | Not original peer-reviewed research | 1 |
| Not peer-reviewed journal article | Not original peer-reviewed research | 1 |
| Conference abstract only | Not original peer-reviewed research | 1 |
| Corrigendum / not original study | Not original peer-reviewed research | 1 |
