# Supplementary material for: Does delayed exercise-based priming improve subsequent athletic performance? A systematic review and multilevel meta-analysis
Source: PLoS One. 2026 Jul 30;21(7):e0354720. doi: 10.1371/journal.pone.0354720 (PMC13422850; doi:10.1371/journal.pone.0354720)
Supplement: S3 Table — (DOCX) [file pone.0354720.s006.docx]

**S3 Table. Characteristics of studies included in the quantitative meta-analysis.**

Intervals are expressed as hours between priming and the subsequent performance outcome. Outcome domains follow the coding used in the meta-analysis dataset.

| Study | Sport | Athlete level | Design | Priming type | Control | Interval | Effects | Outcome domains |
| --- | --- | --- | --- | --- | --- | --- | --- | --- |
| Woolstenhulme (2004) | basketball | competitive | crossover | resistance | passive rest | 6 h | 7 | sport-specific; strength/power |
| Ekstrand (2013) | athletics/throws | trained | crossover | resistance | passive rest | 4 h | 2 | sport-specific; strength/power |
| Cook / Crewther (2014) | rugby | competitive | crossover | sprint-based | passive rest | 6 h | 3 | speed/agility; strength/power |
| Russell (2016) | rugby | competitive | crossover | sprint-based | passive rest | 5 h | 1 | strength/power |
| Mason (2017) | rugby | trained | crossover | resistance | passive rest | 2 h | 2 | strength/power |
| Tsoukos (2018) | power and team sports | trained | crossover | ballistic/jump | passive rest | 24 h | 2 | strength/power |
| Dahl (2021) | running | competitive | crossover | resistance | passive rest | 6 h | 2 | sport-specific; strength/power |
| Donghi (2021) | soccer | elite | crossover | sprint-based | passive rest | 5.5 h | 5 | speed/agility; sport-specific; strength/power |
| Gonzalez-Garcia (2021) | resistance-trained adults | trained | crossover | resistance | passive rest | 6 h | 3 | strength/power |
| Nishioka and Okada (2022) | resistance-trained men | trained | crossover | resistance | passive rest | 24 h | 1 | strength/power |
| Nutt (2022) | cricket | competitive | crossover | sprint-based | passive rest | 5.5 h | 1 | sport-specific |
| Zaras (2022) | swimming | trained | crossover | ballistic/jump | passive rest | 24 h | 2 | sport-specific; strength/power |
| Gonzalez-Garcia (2023) | resistance-trained adults | trained | crossover | resistance | passive rest | 6 h | 1 | strength/power |
| Panteli (2024) | soccer | trained | crossover | ballistic/jump | passive rest | 24 h | 3 | speed/agility; strength/power |
| Wang (2024) | mixed collegiate sports | trained | crossover | resistance | passive rest | 6 h | 3 | speed/agility; strength/power |
| Pino-Mulero (2025) | soccer | competitive | crossover | sprint-based | usual training | 24 h | 2 | speed/agility; strength/power |
| Brisola (2026) | water polo | competitive | crossover | resistance | passive rest | 6 h | 3 | speed/agility; sport-specific; strength/power |
| Kolinger (2026) | soccer | trained | crossover | resistance | low intensity active | 5 h | 3 | speed/agility; sport-specific; strength/power |
