# Supplementary material for: Does delayed exercise-based priming improve subsequent athletic performance? A systematic review and multilevel meta-analysis
Source: PLoS One. 2026 Jul 30;21(7):e0354720. doi: 10.1371/journal.pone.0354720 (PMC13422850; doi:10.1371/journal.pone.0354720)
Supplement: S4 Table — (DOCX) [file pone.0354720.s007.docx]

**S4 Table. Effect-size reconstruction and extractability decisions.**

Note. The table summarizes study-level quantitative compatibility. Most crossover studies did not report paired-condition correlations or paired-difference standard deviations; therefore, repeated-measures SMDs were reconstructed with an assumed within-participant correlation of r = 0.50 in the primary analysis when needed.

| Study | Status | Effects | Effect-size reconstruction | Correlation / variance handling | Reason not pooled |
| --- | --- | --- | --- | --- | --- |
| Woolstenhulme (2004) | Included in quantitative synthesis | 7 | crossover rm-SMD; assumed r=0.5 | Required assumed within-participant correlation (primary r = 0.50; sensitivity r = 0.30, 0.70, 0.90). | NA |
| Ekstrand (2013) | Included in quantitative synthesis | 2 | crossover rm-SMD; assumed r=0.5 | Required assumed within-participant correlation (primary r = 0.50; sensitivity r = 0.30, 0.70, 0.90). | NA |
| Cook et al. (2014) | Included in quantitative synthesis | 3 | crossover rm-SMD; assumed r=0.5 | Required assumed within-participant correlation (primary r = 0.50; sensitivity r = 0.30, 0.70, 0.90). | NA |
| Russell (2016) | Included in quantitative synthesis | 1 | crossover rm-SMD; assumed r=0.5 | Required assumed within-participant correlation (primary r = 0.50; sensitivity r = 0.30, 0.70, 0.90). | NA |
| Mason (2017) | Included in quantitative synthesis | 2 | crossover rm-SMD; assumed r=0.5 | Required assumed within-participant correlation (primary r = 0.50; sensitivity r = 0.30, 0.70, 0.90). | NA |
| Tsoukos (2018) | Included in quantitative synthesis | 2 | crossover rm-SMD; assumed r=0.5 | Required assumed within-participant correlation (primary r = 0.50; sensitivity r = 0.30, 0.70, 0.90). | NA |
| Dahl (2021) | Included in quantitative synthesis | 2 | crossover rm-SMD; assumed r=0.5 | Required assumed within-participant correlation (primary r = 0.50; sensitivity r = 0.30, 0.70, 0.90). | NA |
| Donghi (2021) | Included in quantitative synthesis | 5 | crossover rm-SMD; assumed r=0.5 | Required assumed within-participant correlation (primary r = 0.50; sensitivity r = 0.30, 0.70, 0.90). | NA |
| Gonzalez-Garcia (2021) | Included in quantitative synthesis | 3 | crossover rm-SMD; assumed r=0.5 | Required assumed within-participant correlation (primary r = 0.50; sensitivity r = 0.30, 0.70, 0.90). | NA |
| Nishioka and Okada (2022) | Included in quantitative synthesis | 1 | crossover rm-SMD; assumed r=0.5 | Required assumed within-participant correlation (primary r = 0.50; sensitivity r = 0.30, 0.70, 0.90). | NA |
| Nutt (2022) | Included in quantitative synthesis | 1 | crossover rm-SMD; assumed r=0.5 | Required assumed within-participant correlation (primary r = 0.50; sensitivity r = 0.30, 0.70, 0.90). | NA |
| Zaras (2022) | Included in quantitative synthesis | 2 | crossover rm-SMD; assumed r=0.5 | Required assumed within-participant correlation (primary r = 0.50; sensitivity r = 0.30, 0.70, 0.90). | NA |
| Gonzalez-Garcia (2023) | Included in quantitative synthesis | 1 | crossover rm-SMD; assumed r=0.5 | Required assumed within-participant correlation (primary r = 0.50; sensitivity r = 0.30, 0.70, 0.90). | NA |
| Panteli (2024) | Included in quantitative synthesis | 3 | crossover rm-SMD; assumed r=0.5 | Required assumed within-participant correlation (primary r = 0.50; sensitivity r = 0.30, 0.70, 0.90). | NA |
| Wang (2024) | Included in quantitative synthesis | 3 | crossover rm-SMD; assumed r=0.5 | Required assumed within-participant correlation (primary r = 0.50; sensitivity r = 0.30, 0.70, 0.90). | NA |
| Pino-Mulero (2025) | Included in quantitative synthesis | 2 | reported ES; approximate variance | Used reported effect-size information with approximate variance; no paired correlation assumption used for this study-level coding. | NA |
| Brisola (2026) | Included in quantitative synthesis | 3 | crossover rm-SMD; assumed r=0.5 | Required assumed within-participant correlation (primary r = 0.50; sensitivity r = 0.30, 0.70, 0.90). | NA |
| Kolinger (2026) | Included in quantitative synthesis | 3 | crossover rm-SMD; assumed r=0.5 | Required assumed within-participant correlation (primary r = 0.50; sensitivity r = 0.30, 0.70, 0.90). | NA |
| Saez de Villarreal (2007) | Narrative-only eligible study | 0 | Not converted to standardized mean difference | Not applicable; insufficient compatible variance information for rm-SMD. | Percent change only; no usable SD or change SD for SMD. |
| McGowan (2017) | Narrative-only eligible study | 0 | Not converted to standardized mean difference | Not applicable; insufficient compatible variance information for rm-SMD. | Primary time-trial outcome reported as percent change/CI; not converted for main SMD. |
| Harrison (2021) | Narrative-only eligible study | 0 | Not converted to standardized mean difference | Not applicable; insufficient compatible variance information for rm-SMD. | Percent baseline/CI style reporting without raw mean/SD. |
| Rud (2021) | Narrative-only eligible study | 0 | Not converted to standardized mean difference | Not applicable; insufficient compatible variance information for rm-SMD. | Percent difference +/- 95% CI only; no raw mean/SD. |
| Holmberg (2026) | Narrative-only eligible study | 0 | Not converted to standardized mean difference | Not applicable; insufficient compatible variance information for rm-SMD. | LMM marginal means and MD 95% CI; no raw SD for SMD. |
| Harrison (2024) | Narrative-only eligible study | 0 | Not converted to standardized mean difference | Not applicable; insufficient compatible variance information for rm-SMD. | Percent of control plus Cliff's delta; no raw mean/SD. |
| Harrison (2023) | Narrative-only eligible study | 0 | Not converted to standardized mean difference | Not applicable; insufficient compatible variance information for rm-SMD. | Percent of control plus Cliff's delta; no raw mean/SD. |
| Gonzalez-Garcia caffeine (2023) | Narrative-only eligible study | 0 | Not converted to standardized mean difference | Not applicable; insufficient compatible variance information for rm-SMD. | Percent of baseline plus Cohen's d; no raw mean/SD per condition. |
