# Supplementary material for: Does delayed exercise-based priming improve subsequent athletic performance? A systematic review and multilevel meta-analysis
Source: PLoS One. 2026 Jul 30;21(7):e0354720. doi: 10.1371/journal.pone.0354720 (PMC13422850; doi:10.1371/journal.pone.0354720)
Supplement: S5 Table — (DOCX) [file pone.0354720.s008.docx]

**S5 Table. GRADE summary of findings.**

GRADE = Grading of Recommendations Assessment, Development and Evaluation; PI = prediction interval. Certainty ratings summarize the overall confidence in the effect estimate for each analysis and should be interpreted with the exploratory nature of subgroup analyses in mind.

| Outcome or analysis | Studies | Effect sizes | Effect estimate | Main reason for downgrade | Certainty |
| --- | --- | --- | --- | --- | --- |
| Overall athletic performance | 18 | 46 | g = 0.232 (95% CI 0.092 to 0.372); PI -0.265 to 0.730 | Serious inconsistency/contextual variability; crossover reporting concerns | Low |
| Direct mean/SD-only evidence | 13 | 37 | g = 0.105 (95% CI 0.005 to 0.205) | Imprecision and reduced extractable evidence base | Low |
| Strength/power outcomes | 17 | 26 | g = 0.212 (95% CI 0.097 to 0.326) | Some risk-of-bias concerns and imprecision | Low |
| Speed/agility outcomes | 7 | 9 | g = 0.377 (95% CI -0.025 to 0.778) | Inconsistency and imprecision | Very low |
| Sport-specific outcomes | 8 | 11 | g = 0.075 (95% CI -0.117 to 0.268) | Indirectness and imprecision | Very low |
| Intervals of at least 6 h | 12 | 32 | g = 0.288 (95% CI 0.086 to 0.490); PI -0.348 to 0.924 | Serious inconsistency/contextual variability | Low |
