# Supplementary material for: Does delayed exercise-based priming improve subsequent athletic performance? A systematic review and multilevel meta-analysis
Source: PLoS One. 2026 Jul 30;21(7):e0354720. doi: 10.1371/journal.pone.0354720 (PMC13422850; doi:10.1371/journal.pone.0354720)
Supplement: S6 Table — (DOCX) [file pone.0354720.s009.docx]

**S6 Table. Leave-one-study-out sensitivity analysis.**

Note. Each row shows the pooled study-level random-effects estimate after omitting one study from the quantitative synthesis, recalculated in R using metafor with REML estimation and Knapp-Hartung inference. Positive Hedges' g indicates better performance after delayed priming.

| Omitted study | Pooled g after omission | 95% CI lower | 95% CI upper | tau2 | I2 (%) |
| --- | --- | --- | --- | --- | --- |
| Ekstrand (2013) | 0.241 | 0.080 | 0.402 | 0.061 | 70.8 |
| Cook et al. (2014) | 0.226 | 0.064 | 0.388 | 0.061 | 70.0 |
| Russell (2016) | 0.224 | 0.066 | 0.382 | 0.058 | 70.2 |
| Mason (2017) | 0.243 | 0.083 | 0.403 | 0.061 | 70.6 |
| Tsoukos (2018) | 0.219 | 0.060 | 0.377 | 0.057 | 69.2 |
| Donghi (2021) | 0.243 | 0.082 | 0.405 | 0.062 | 69.8 |
| Dahl (2021) | 0.242 | 0.082 | 0.402 | 0.060 | 70.7 |
| Zaras (2022) | 0.239 | 0.077 | 0.400 | 0.062 | 70.9 |
| Gonzalez-Garcia (2021) | 0.229 | 0.068 | 0.391 | 0.062 | 70.7 |
| Nutt (2022) | 0.238 | 0.077 | 0.398 | 0.061 | 71.0 |
| Nishioka and Okada (2022) | 0.240 | 0.079 | 0.400 | 0.061 | 70.9 |
| Panteli (2024) | 0.239 | 0.077 | 0.401 | 0.063 | 70.7 |
| Wang (2024) | 0.198 | 0.052 | 0.344 | 0.041 | 61.3 |
| Woolstenhulme (2004) | 0.261 | 0.109 | 0.413 | 0.047 | 61.6 |
| Pino-Mulero (2025) | 0.183 | 0.071 | 0.294 | 0.026 | 50.8 |
| Brisola (2026) | 0.241 | 0.080 | 0.403 | 0.062 | 70.4 |
| Kolinger (2026) | 0.251 | 0.093 | 0.409 | 0.057 | 68.7 |
| Gonzalez-Garcia (2023) | 0.244 | 0.086 | 0.402 | 0.059 | 70.4 |
